# Electrochemically Induced Nanoscale Stirring Boosts Functional Immobilization of Flavocytochrome P450 BM3 on Nanoporous Gold Electrodes

**DOI:** 10.1002/smtd.202400844

**Published:** 2024-09-19

**Authors:** Elisabeth Hengge, Eva‐Maria Steyskal, Alexander Dennig, Manfred Nachtnebel, Harald Fitzek, Roland Würschum, Bernd Nidetzky

**Affiliations:** ^1^ Institute of Biotechnology and Biochemical Engineering Graz University of Technology Petersgasse 12 Graz 8010 Austria; ^2^ Institute of Materials Physics Graz University of Technology Petergasse 16 Graz 8010 Austria; ^3^ Graz Centre for Electron Microscopy (ZFE) Steyrergasse 17 Graz 8010 Austria; ^4^ Austrian Centre of Industrial Biotechnology (acib) Petersgasse 14 Graz 8010 Austria

**Keywords:** bioelectrochemistry and biosensing, biointerface engineering, charge‐directed functional immobilization, enzyme electrode, flavocytochrome P450, nanoporous gold, self‐assembled monolayer

## Abstract

Enzyme‐modified electrodes are core components of electrochemical biosensors for diagnostic and environmental analytics and have promising applications in bioelectrocatalysis. Despite huge research efforts spanning decades, design of enzyme electrodes for superior performance remains challenging. Nanoporous gold (npAu) represents advanced electrode material due to high surface‐to‐volume ratio, tunable porosity, and intrinsic redox activity, yet its coupling with enzyme catalysis is complex. Here, the study reports a flexible‐modular approach to modify npAu with functional enzymes by combined material and protein engineering and use a tailored assortment of surface and in‐solution methodologies for characterization. Self‐assembled monolayer (SAM) of mercaptoethanesulfonic acid primes the npAu surface for electrostatic adsorption of the target enzyme (flavocytochrome P450 BM3; CYT102A1) that is specially equipped with a cationic protein module for directed binding to anionic surfaces. Modulation of the SAM surface charge is achieved by electrochemistry. The electrode‐adsorbed enzyme retains well the activity (33%) and selectivity (complete) from in‐solution. Electrochemically triggered nanoscale stirring in the internal porous network of npAu‐SAM enhances speed (2.5‐fold) and yield (3.0‐fold) of the enzyme immobilization. Biocatalytic reaction is fueled from the electrode via regeneration of its reduced coenzyme (NADPH). Collectively, the study presents a modular design of npAu‐based enzyme electrode that can support flexible bioelectrochemistry applications.

## Graphical abstract

A flexible‐modular approach for enzyme immobilization on nanoporous gold (npAu). A cationic protein module for directed binding is fused to the enzyme. The surface is modified with self‐assembled monolayer. Surface charge is modulated electrochemically. Thereby induced nanoscale stirring in the porous network strongly enhances yield and speed of enzyme adsorption. Regeneration of coenzyme on npAu enables versatile applications in bioelectrochemistry.

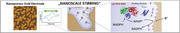

## 1 Introduction

Enzymes are exceptionally efficient catalysts of chemical reactions and achieve their transformations with exquisite specificity. To couple enzymes with electrodes for joint cooperative function represents a longstanding central aim in the field of bioelectrochemistry and its allied technologies.^[^ ^1^, ^2^, ^3^ ^]^ Enzyme‐modified electrodes are functional devices based on a composite material that allows for connecting a selective biocatalytic reaction to an electron‐transfer process at a solid surface. The role of the electron transfer varies from promoting the enzymatic reaction, as in bioelectrocatalysis, to electrochemical detection, as in analytical sensing.^[^ ^4^, ^5^ ^]^ Despite the immense interest that enzyme electrodes have drawn in fundamental and use‐inspired studies alike, their development for robust function in bioelectrochemical applications still remains challenging. Major limitations arise due to issues of efficiency (i.e., activity, sensitivity) and operational stability.^[^ ^5^ ^]^ Both issues typically have their mechanistic origin in unsuitable characteristics of the enzyme interaction with the solid surface of the plain electrode. Addressing these issues through an advanced design of enzyme electrode necessitates an integrative engineering approach that considers the material, the surface, and the enzyme alike. Here we present such an approach on the basis of nanoporous gold (npAu) electrodes.

npAu is particularly promising as electrode material due to the high surface‐to‐volume ratio, the tunable pore size, and the high activity of electron transfer that it represents.^[^ ^6^, ^7^, ^8^ ^]^ Studies on enzymatic‐npAu biosensors^[^ ^9^, ^10^, ^11^, ^12^ ^]^ and biofuel cell electrodes^[^ ^13^, ^14^ ^]^ highlight its great potential. npAu can be produced by electrochemical etching (“dealloying”) of an AuAg master alloy whereby the less noble Ag is dissolved and the remaining Au forms a bicontinuous porous structure.^[^ ^15^, ^16^, ^17^, ^18^ ^]^ When applying bulk, rigid nanoporous samples as electrode material, accessibility of the pore‐internal surface^[^ ^19^ ^]^ and speed of binding limited by diffusion can result in low performance efficiency. Furthermore, standard microscopic or spectroscopic techniques are usually inadequate to assess the internal surface.^[^ ^20^, ^21^ ^]^ In previous work,^[^ ^22^ ^]^ we have therefore optimized the npAu fabrication to adjust the pore size to the requirements of the desired application and have established electrochemical impedance spectroscopy (EIS) as means of analyzing the bonding of single‐molecule layers on the npAu surface. In the present work, we significantly extend the EIS methodology to also analyze the additional binding of proteins on nanoporous electrodes and in doing so, we address a major analytical challenge of enzyme electrode development in general.

The plain Au surface is not well suited for enzyme immobilization due to low protein binding and protein denaturation often associated with the adsorption.^[^ ^23^ ^]^ However, its modification through monolayer self‐assembly of thiol group‐containing molecules is powerful to overcome these limitations. The molecular “head groups” of the self‐assembled monolayer (SAM) attached via sulfur‐metal covalent bond provide flexibility to tune the physical and electrochemical properties of the surface to the requirements of functional enzyme immobilization.^[^ ^24^, ^25^ ^]^ Working with carboxyl group‐terminated SAMs on npAu, we have shown recently that electrochemical modulation of the head group ionization provides additional means of controlling the surface charge.^[^ ^22^ ^]^ The current study was designed to exploit the electrochemically inducible polarization of the SAM for enhanced enzyme immobilization on npAu electrodes. In particular, electrochemical “nanoscale stirring” is induced in the porous structure, thereby modulating the mutual interaction between enzyme and electrode.

**Figure 1:**
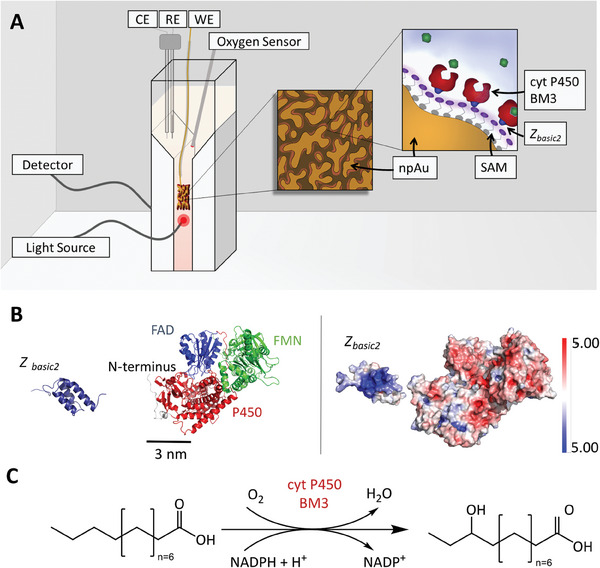
Enzyme electrode design exploiting the Z_basic2_ module fused to BM3. A) Proposed structure of the enzyme electrode and set‐up for its characterization. RE‐reference electrode, CE‐counter electrode, WE‐working electrode. B) Structure of BM3 (AlphaFold entry: P14779) consisting of the P450‐heme, FMN, and FAD domains, and Z_basic2_ module (left) as well as its surface charge distribution (right), red – negative, blue – positive. Protein structures are visualized with The PyMOL Molecular Graphics System, Version 2.6 Schrödinger, LLC. Surface charge was calculated and color scale‐represented by APBS software.^[^ ^43^ ^]^ C) BM3 reaction with lauric acid.^[^ ^26^, ^44^ ^]^

In devising our strategy to enzyme electrode development, we considered flexible application to different enzymes as an important goal. The concept of fusion protein builds on the idea of modular protein organization to facilitate exchange of the component parts. In the context of immobilization on electrodes, a unique protein module for solid adsorption is linked genetically to the enzyme of interest which can be variable. Based on our earlier research,^[^ ^26^, ^27^, ^28^, ^29^ ^]^ we considered the cationic module Z_basic2_ (p*I* = 10.5^[^ ^30^ ^]^) to promote an orientationally directed adsorption of fusion proteins to anionic surfaces. The Z_basic2_ is a small protein unit (58 amino acids) that folds into a stable α‐helical bundle structure.^[^ ^30^ ^]^ Surface binding of Z_basic2_ is driven electrostatically by accumulation of positive charges from surface‐exposed arginines. Immobilization studies on carrier particles showed that the sulfonate group was particularly effective in giving strong interaction with Z_basic2_ fusion proteins.^[^ ^31^ ^]^ Considering these evidences, we selected mercaptoethanesulfonic acid (MESA) for SAM formation on npAu to adsorb enzyme fused to Z_basic2_. Due to the short alkyl chain of MESA, the resulting SAM might be amenable well to electrochemical polarization, with detrimental effects on capacitance (known for longer‐chain SAMs) avoided. The conceptual approach is depicted in **Figure** **1** and we demonstrate it herein for the well‐known flavocytochrome P450 BM3 (CYT102A1; henceforth referred to as BM3 for simplicity).^[^ ^32^ ^]^ Our previous work has shown the Z_basic2_ fusion of BM3 for functional immobilization on sulfonate carriers.^[^ ^26^ ^]^

BM3 is a monoxygenase that inserts oxygen from O_2_ into unactivated C‐H bonds in diverse substrates, including simple fatty acids as shown in Figure 1C. The hydroxylation by BM3 requires two electrons to activate the O_2_ for the chemistry performed. These electrons are usually supplied from NADPH coenzyme and are shuttled via flavin to heme iron in the enzyme's catalytic center (from FAD via FMN to the P450 domain, see Figure 1B). The second atom of the O_2_ substrate is released as water (Figure 1C). Coupling efficiency of BM3 refers to the portion of O_2_ reduced by NADPH ending up in the hydroxylated product. With a “perfect” value of unity, the coupling efficiency can vary in a broad range (≤1) depending on the BM3 preparation (e.g., immobilized enzyme) and the conditions used. The uncoupled reaction releases H_2_O_2_.

BM3 represents a large class of flavocytochrome P450 oxidoreductases that have attracted major interest for enzyme electrode development. Their involvement in drug metabolism and hormone regulation makes P450 enzymes important candidates for clinical biosensing. BM3 has been studied on diverse electrode materials including mesoporous silica nanoparticles on a glassy carbon electrode,^[^ ^33^ ^]^ on gold films functionalized with maleimide‐terminated SAMs,^[^ ^34^ ^]^ on modified mesoporous tin‐oxide coated glass^[^ ^35^ ^]^ as well as on modified^[^ ^36^, ^37^ ^]^ and unmodified graphite electrodes^[^ ^38^ ^]^ (see also Valikhani et al.^[^ ^39^ ^]^ for comprehensive review on P450 functional immobilization).

Their mastery of catalysis to selective C‐H activation, which is among the most difficult transformations to be performed chemically, makes the P450 enzymes attractive for synthesis. Coupling of the enzymatic electron transfer to the electrode can happen in two ways: mediated via the NADP^+^/NADPH in solution or directly from the enzyme, typically via the heme‐iron. Both types of electron transfer involve major issues of practical efficiency and selectivity (e.g., uncatalyzed formation of inactive nicotinamide dimers). Diverse nanostructured electrodes (such as deposited nanoparticles or carbon composites) have proven to catalyze the electrochemical reduction of NAD(P)^+^.^[^ ^40^, ^41^, ^42^ ^]^ Here, we demonstrate it on the nanoporous gold electrode opening potential application as functional biosensors.

## 2 Tailoring the Nanoporosity and the Surface Character of npAu

Free‐standing, bulk AuAg platelets with dimension of 500 × 500 × 150 µm were used to prepare npAu by electrochemical dealloying.^[^ ^18^, ^20^, ^45^ ^]^ As‐dealloyed samples show pore sizes in the range 10–20 nm. Considering pore size requirements for efficient infusion of enzymes into the pore network, we here used isothermal coarsening^[^ ^46^ ^]^ to prepare of npAu with substantially enlarged pores (≥100 nm, see later). Of note, the hydrodynamic radius of the active BM3 homodimer^[^ ^47^, ^48^ ^]^ is ∼6 nm,^[^ ^49^ ^]^ emphasizing the need for pore sizes well above 20 nm to avoid pore clogging by just a single event of enzyme adsorption. Importantly, annealing at elevated temperatures entails increased stability at room temperature, as required for further use as electrode. The resulting pore size determined electrochemically^[^ ^50^, ^51^ ^]^ (see Supplementary Methods and Figure S1, Supporting Information) increased strongly with the annealing temperature used (Figure S2, Supporting Information), consistent with the literature.^[^ ^52^ ^]^ While its exact atomistic description remains elusive, the dealloying process generally shows self‐similar coarsening of pores and ligaments accompanied by surface reduction, thereby retaining the bicontinuous structure. The Arrhenius dependence (Figure S2, Supporting Information inset) supports the notion of a surface‐diffusion driven process^[^ ^53^ ^]^ and the calculated apparent activation energy of 0.21 eV is in line with the literature.^[^ ^54^ ^]^ Based on these advances, we were now able to tailor the nanoporosity of the npAu base material for the subsequent BM3 immobilization.

To monitor formation of the MESA‐SAM on npAu, we used in situ resistometry based on method developed in our earlier work.^[^ ^55^ ^]^ Due to the small dimensions of the ligaments, resistance of npAu is mainly dependent on electron scattering at the Au surface. Changes in chemical composition such as chemisorption of thiol‐groups on the gold surface increases the total resistance of the sample. As shown in Figure S3 (Supporting Information), the adsorption process of MESA causes a relative increase of resistance by 10% and required at least 48 h to complete. Subsequently, 48 h immersion time was used for npAu support fabrication for BM3 immobilization. The long duration of the adsorption process is caused by slow diffusion into the rigid, porous network and the complex assembly process on the nanostructured surface.^[^ ^55^ ^]^ By correlating the change transferred during electrochemical desorption of MESA (reduction of Au‐S) to the concomitant resistance decrease, an initial surface coverage of 0.7 monolayer was determined (see also Supplementary Methods and Figure S3, Supporting Information).

**Figure 2:**
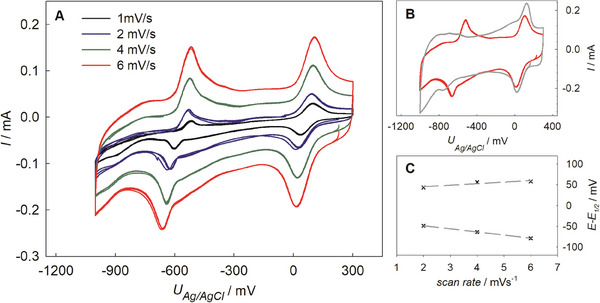
Electrochemistry of npAu/MESA. A) CVs in 50 mm KPi buffer (pH 8) at varying scan rates (see legend). B) Grey: CVs of pristine npAu sample (reference) and red: reprinted from (A); both 6 mVs^−1^. C) Laviron‐plot (E‐E_1/2_ vs scan rate) deduced from the experimental data presented in (A).

Cyclic voltammograms (CVs) of MESA‐modified npAu (henceforth referred to as npAu/MESA) reveal two redox peak couples (**Figure** **2**A). The peaks between +150 and 0 mV arise from npAu surface as revealed by reference measurements (Figure 2B) and are presumably caused by the residual Ag in the porous structure.^[^ ^56^ ^]^ The observed Ag content is expected to play a minor role in the further analysis of the electrode as these peaks are well separated from the potential range of interest. Moreover, studies of SAM formation on Ag and Au reveal comparable behavior.^[^ ^57^, ^58^ ^]^ The second peak couple lies between −520 and ‐660 mV with the mid‐potential value E_1/2_ = −580 mV and originate from the MESA‐layer. The shift of peak positions as a function of scan rate suggests a surface‐confined redox reaction^[^ ^59^ ^]^ and strongly hints toward a protonation/deprotonation process. The degree of deprotonation of SAMs is often wrongly assumed invariant upon varying potential (at constant pH). Comprehensive series of publications^[^ ^60^, ^61^, ^62^, ^63^ ^]^ unveil this electric‐field driven (pseudo‐capacitive) proton transfer reaction at the terminal group of the SAM, mainly focusing on carboxylic groups. In contrary, electrochemical protonation/deprotonation of sulfonate groups is less investigated, yet was observed for MESA in an early work by Fawcett et al.^[^ ^63^ ^]^ Under the here applied conditions, electrochemical induced protonation was unexpected as their solution‐state p*K*_a_^[^ ^64^ ^]^ lies between −5 and −6 and thus several orders lower than that of carboxylic acid groups. However, it is generally known that the apparent surface p*K*_a_ of functional groups can vary greatly depending on nature of the carrier and electrolyte. For carboxylic groups, p*K*_a_ ranging from 4 to 10 have been reported.^[^ ^65^, ^66^, ^67^ ^]^ For surface‐confined sulfonate groups a p*K*_a_ of 2 was shown by Shyue et al.^[^ ^64^ ^]^ Importantly, the assembly (ordering) of SAMs strongly affects the proton transfer reaction and its voltammetric response.^[^ ^59^, ^62^ ^]^ In previous work,^[^ ^22^ ^]^ we discussed the beneficial effects of disordered assembly caused by the intrinsic surface roughness and curvature of npAu. Similar can be assumed for MESA. All these studies taken together strongly support peak assignment to sulfonate protonation/ deprotonation.

The assessment of nanoporous metal electrodes is complex. However, to estimate the proton transfer frequency, one can follow the approximation by Luque et al.^[^ ^62^ ^]^ based on a model for simple charge transfer at strongly adsorbed redox species (see Hengge et al.^[^ ^22^ ^]^). From the peak separation (E‐E_1/2_) and its dependence on the scan rate (Laviron plot, Figure 2C), the electron transfer rate was roughly approximated as 3 × 10^2^ s^−1^ which is in line with literature for carboxylic group‐containing SAMs.^[^ ^22^, ^59^ ^]^ Following the same approximation, correlation of the total transferred charge during protonation to the surface coverage reveals that roughly 5% of molecules participate in this reaction. This value must be taken with caution as such, however it indicates only partial protonation upon electrochemical potential control.

Summing up, under the conditions applied for the immobilization of BM3, the MESA is negatively charged on surfaces (yet significantly less than in‐solution) and hence can attract positively charged biomolecules for adsorption. It is further hypothesized that concurrently, by changing the degree of protonation electrochemically, the mutual electrostatic interactions between the surface sulfonate groups and the Z_basic2_ module can be altered.

## 3 Electrostatic Adsorption of BM3 to npAu/MESA

### 3.1 Biochemical Characterization of the Immobilization

Absorption spectroscopy (based on the specific absorption peak of the heme group in BM3 at 420 nm) was adopted as a sensitive and selective spectroscopic method to monitor the immobilization process. Change in enzyme concentration in the supernatant over time was measured. As shown in Figure S4 (Supporting Information), absorption decrease relative to the reference (enzyme solution without npAu) indicates binding of the enzyme to the npAu/MESA. Interestingly, the ongoing decrease lasts up to 50 h (Figure S4, Supporting Information). The adsorption of BM3 on npAu/MESA is significantly slower than on other porous carriers (e.g., Bolivar et al.^[^ ^28^ ^]^). We explain this phenomenon by the high tortuosity of the channels in the monolithic samples and the slow transport (infusion) of enzyme into the pores.^[^ ^19^ ^]^ For practical reasons and to avoid significant enzyme activity loss over time, an incubation time of 15 h (900 min) was chosen to assemble the electrode. According to Figure S4 (Supporting Information), the time used corresponds to 50% of the total absorption achieved after 50 h.

#### 3.1.1 Immobilization Yield and Surface Density of Adsorbed BM3

**Figure 3:**
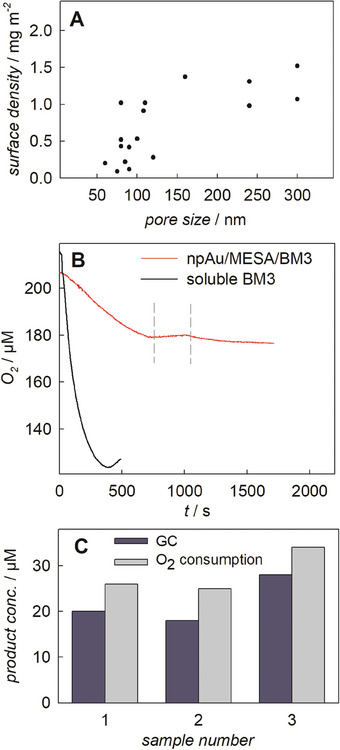
Electrostatic immobilization of BM3 on npAu/MESA. A) Dependence of the surface density in mg m^‐^^2^ on the pore size of npAu. B) O_2_ consumption during the hydroxylation of lauric acid using the npAu/MESA/BM3 electrode (red) as catalyst. Removing the electrode stops the reaction (dashed vertical lines) and placing it back initiates the reaction again. Black: Reference using 200 µL soluble BM3. Reaction conditions: 200 µm lauric acid, 200 µm NAPDH in 50 mm KPi buffer (total volume of 2.0 mL), 30 °C, magnetically stirred at 300 rpm. C) Comparison of final product concentration (hydroxylauric acid) deduced from O_2_ consumption and quantitative GC analysis. See Figure S5 (Supporting Information) for chromatogram of (hydroxy‐)lauric acid.

The surface density (given in mg_enzyme_ adsorbed/cm^2^) as a measure of the enzyme coverage is best suited to characterize the pore size dependence of the enzyme immobilization. The adsorbed enzyme (per unit volume) is determined from the enzyme concentration difference in solution before and after immobilization. The npAu/MESA surface area (per unit volume) is known from the previous material characterization and the carrier mass used in the immobilization experiment. Pores of up to roughly 80 nm result in very low coverage (**Figure** **3**A). Agglomeration (i.e., formation of multilayer islands of aggregated enzymes) probably causes pore clogging upon absorption of BM3. For pore sizes in the range 80–100 nm, the surface density is ≈0.6 mg m^−2^. For reference, immobilization on similar samples without SAM surface modification only results in 0.09 mg m^−2^ surface coverage. Using npAu/MESA samples with 100 nm pore size, 38 ± 3 µg mL^−1^ of the purified BM3 was immobilized by loading 110 ± 10 µg mL^−1^ (equal to 0.85 ± 0.07 µm, N = 3) which corresponds to an immobilization yield of 36%. This electrode preparation exhibits good reproducibility: standard deviation in enzyme loading lies within 8%. Upon storage at 4 °C in buffer solution no leakage was observed for at least 4–5 h (data not shown). The immobilization data on npAu/MESA are used to calculate a specific binding of 1.4 ± 0.3 mg/g_carrier_ which can be compared to a value of 10–13 mg/g_carrier_ for immobilization of the same Z_basic2_ fusion of BM3 as used here on spherical polymethacrylate particles with sulfonate surface groups (see earlier works Valikhani et al.^[^ ^26^ ^]^ and Buergler et al.^[^ ^44^ ^]^). Considering material density of polymethacrylate (1.2 g cm^−3^)^[^ ^68^ ^]^ and gold (19.3 g cm^−3^)^[^ ^69^ ^]^ these values are in good agreement.

Results in Figure 3A furthermore show that on increase of the npAu pore size the enzyme surface coverage increases up to 1.0–1.2 mg m^−2^. Saturation is reached for pore sizes of 150 nm and higher, examined up to 300 nm. Using npAu/MESA of roughly 270 nm pore size, 27 ± 6 µg mL^−1^ was immobilized with a lowered yield of 25% (N = 3, BM3 stock solution 120 ± 50 µg mL^−1^). Comparison of the yield and surface density on npAu/MESA with ≈100 and ≈270 nm show opposite trends with pore size, explainable by the fact that the coarsened nanoporous sample involves an intrinsically smaller total surface area. For all subsequent studies, npAu/MESA samples with ≈300 nm pore size were used as higher enzyme surface density is expected to have better functional properties.

#### 3.1.2 Retained BM3 Activity and Selectivity

To assess the activity of immobilized BM3, we measured the conversion of lauric acid to hydroxylauric acid (reaction is shown in Figure 1C). A reaction time course based on O_2_ consumption is exemplarily shown in Figure 3B. Based on comparison to the soluble enzyme the immobilized BM3 retains 33% (±1%, N = 3) of the original activity. Considering the multidomain structural complexity of the functional BM3 and the diffusion limitation in the porous structure, this level of activity retention is substantial, and it can probably be ascribed to the productive interplay between tailored surface modification and oriented immobilization via the Z_basic2_ module. The BM3 activity on npAu/MESA is only slightly lower than it is on sulfonate polymethacrylate carrier particles (48% retention, see previous work^[^ ^26^ ^]^). The lowering of the BM3 activity in the immobilized state can arise due to various reasons which to explore more fully was beyond the scope of the current study. Considering diffusional effects, the value of 33% of retained activity in the immobilized BM3 should be interpreted as an apparent activity. We are also aware of the important role of macroscopic geometry of electrode for the optimization of performance efficiency. Important earlier works have highlighted the use of 3D‐printed scaffolds or hierarchically organized material structures (see review by Wittstock et al.^[^ ^19^ ^]^). At this point, however, we content ourselves with one single sample geometry.

Figure 3B also shows that the O_2_ consumption by npAu/MESA/BM3 is strictly due to the adsorbed BM3. Removal of the electrode stops the reaction. The slight increase in the soluble O_2_ concentration during the phase of removed electrode is due to the low O_2_ transfer from air. Upon reinserting of the electrode, the reaction continues.

As explained in the Introduction, coupling efficiency (= molar ratio of substrate hydroxylated/NADPH consumed for O_2_ reduction) is a key parameter of BM3 reaction selectivity.^[^ ^44^ ^]^ As BM3 coupling efficiency can change on immobilization,^[^ ^70^ ^]^ we determined it for the npAu/MESA/BM3 preparation with a value of 77% (±4%, N = 3). It is calculated via measurement of O_2_ consumption and hydroxylauric acid formation (by means of gas chromatography), see Figure 3C for results. The purified BM3 in solution shows a coupling efficiency of 79%, as shown in our previous work.^[^ ^44^ ^]^ We note that retention of coupling efficiency is profoundly important for effectively all applications of BM3 electrode platforms, whether it be in biosensing or bioelectrocatalysis.

### 3.2 Surface Characterization of the npAu/MESA/BM3 Electrode

#### 3.2.1 Electrochemical Impedance Spectroscopy (EIS)

**Figure 4:**
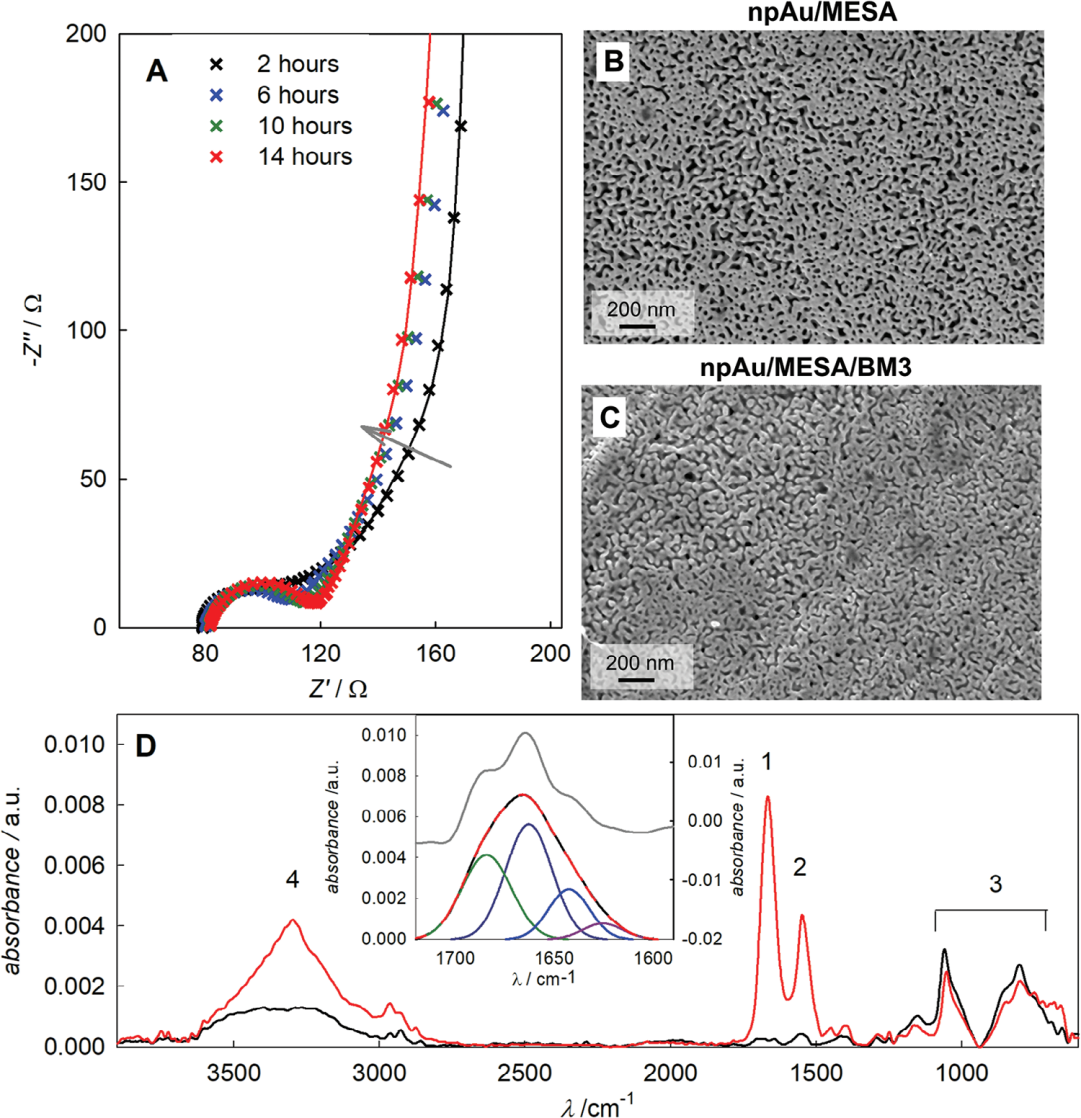
Characterization of npAu/MESA/BM3. A) In situ monitoring of immobilization of BM3 on npAu/MESA by EIS. Spectra were recorded every 2 h for a total of 15 h. The first spectrum was recorded after 2 h to ensure a stable signal. Initial concentration: 0.70 µm, final concentration: 0.52 µm (in 50 mm KPi, pH 7.5). For the sake of clarity, only every second spectrum is plotted. The grey arrow indicates the shift of spectra over time. The times given refer to starting point of each spectrum. B,C) SEM images of (B) npAu/MESA and (C) npAu/MESA/BM3 recorded at the cross‐section of freeze‐dried and halved electrodes. D) GIR‐FTIR‐spectra of planar gold reference samples. Red: npAu/MESA/BM3, black: npAu/MESA. Inset: Background corrected difference spectra of the amide I peak (black), self‐deconvoluted peak in grey and fit components: purple (1663 cm^−1^), green (1684 cm^−1^), blue (1643 cm^−1^), and magenta (1626 cm^−1^) as well as the cumulative peak shown in red (dashed line).

To advance the characterization of npAu/MESA/BM3 beyond the capabilities of in‐solution methods of biochemistry, we here adopted EIS. Known as a highly versatile method to study the electrode‐electrolyte interface, EIS was promising to characterize the BM3 immobilization, in a noninvasive manner and with temporal resolution. We considered that the immobilized enzyme represents an additionally (partially covering) insulating layer^[^ ^71^ ^]^ on the npAu/MESA surface, implying that enzyme adsorption would be recognized in the impedance spectra. Results are shown in **Figure** **4**A.

The temporal evolution of impedance spectra reveals a shift in both the mid‐ and low‐frequency regime. The mid‐frequency regime correlates to a change of the apparent pore shape.^[^ ^71^ ^]^ Agglomeration of proteins during infusion of the porous structure can well explain this change in apparent morphology. In the low‐frequency regime (toward high Z_0_ values), a small shift in the phase angle occurs, as visible by the vertical alignment of data points (Figure 4A). This indicates a change in the capacitive contribution over time, correlating to a protein layer formation on the surface.

The approach of electrical equivalent circuit (EEC) to fit the impedance data is based on a (truncated) transmission line for porous electrodes by de Levie^[^ ^72^ ^]^ and Keiser^[^ ^73^ ^]^ (Figure S6 and Table S1, Supporting Information). Application of EEC approach to SAM‐modified nanoporous electrodes has been discussed in our previous publication.^[^ ^22^ ^]^ Here, following report of Xie et al.,^[^ ^74^ ^]^ we add one additional RC element to the EEC which is used to represent the protein layer. The EEC fit results describe the experimental data very well (Figure 4A). It must be noted that no unique equivalent circuit exists for nanoporous metal electrodes, so further separation of the contributions from SAM, adsorbed enzyme, and porous structure is not scientifically meaningful.

Immobilization yield (30%) and protein surface density of 1.3 mg m^−2^ calculated from the supernatant are well in line with the respective values for immobilization without in situ EIS indicating that the immobilization kinetics remain unaffected upon in situ EIS. EIS has been applied to the qualitative and quantitative analysis of protein adsorption on several types of electrodes.^[^ ^75^, ^76^ ^]^ However, in contrary to the present study, earlier works have typically added inorganic redox‐active species to the electrolyte (e.g., [Fe(CN)_6_]^3−/4−^). Changes in the electron transfer kinetics during the faradaic reactions are then correlated to the adsorption of protein or the formation of a biomolecule layer.^[^ ^74^, ^76^ ^]^ In the pure blocking conditions used in our EIS measurements (i.e., no faradaic reactions occur at the interface), we propose that any change in the interfacial capacitance must be related to a surface protein layer and may thus be used to monitor its formation.

#### 3.2.2 Scanning Electron Microscopy (SEM)

Based on contrast and image sharpness, SEM can identify organic layers formed on npAu surfaces.^[^ ^77^ ^]^ To exclude effects of sample heterogeneity among individually prepared npAu/MESA samples, we used a single sample and separated it into two pieces immediately before the immobilization. Differences in the SEM images of npAu/MESA/BM3 sample (Figure 4C) and reference (Figure 4B) are therefore due to the immobilization of BM3. SEM images were recorded from the cross‐section of halved samples to directly analyze the center of the bulk sample (Figure S7, Supporting Information). Indeed, the SEM image of the immobilized sample involves areas of varying contrast, especially in the upper half of the image, that are not observed in the reference image. These bright regions are interpreted as protein layer (appearing as agglomerates or islands) on top of a metallic surface. The findings agree with the evidence from EIS that correlated the apparent change in pore shape to an inhomogeneous agglomeration of the proteins. Hence, the SEM results confirm the binding of BM3 on the porous structure and show penetration of the entire sample. From the enzyme surface density determined in Section 3.1 and the hydrodynamic radius of BM3,^[^ ^49^ ^]^ a theoretical (homogeneous) total coverage of 1–2 monolayer can be estimated, well in line with the results of microscopic characterization.

#### 3.2.3 Grazing Incidence Reflectance Fourier Transform Infrared (GIR‐FTIR)

We applied GIR‐FTIR to examine structural properties of the immobilized BM3 (for general review, see ref. [78, 79]). Considering the requirement of GIR‐FTIR for a planar solid surface, we used two planar gold samples and modified one with MESA and the other with MESA/BM3. The obtained spectra (Figure 4D) are analyzed based on literature data as follows. The two peaks (#3) present in both samples are assigned to the SO_3_‐groups of the SAM.^[^ ^80^ ^]^ The peak #4 is assigned to the primary and tertiary amide stretching vibration (N‐H) in the protein chain.^[^ ^79^, ^81^ ^]^ Peaks #1 and #2 are assigned to the Amide I and Amide II region of IR spectra.^[^ ^79^, ^81^ ^]^ They arise from the C = O stretching and N‐H bending (coupled with CN stretching) from the amide bonds in the protein. The Amide I peak is commonly used to infer protein secondary structure and hence draw conclusions on the protein conformational states/changes adopted on binding on the surface. After subtraction of the reference spectra (accounting for the signal of the underlying SAM) followed by baseline correction, the Amide II peak was fitted with four Gaussian peaks, as shown in the inset of Figure 4D. The number of peaks were deduced from the Fourier self‐deconvoluted Amide II peak (shown in grey in the inset), which sharpens the overlapping peaks.^[^ ^82^ ^]^ The four band components are assigned to the α‐helix (1663 cm^−1^), the β‐turns (1684 cm^−1^), random coils (1643 cm^−1^), and β‐sheets (1626 cm^−1^).^[^ ^79^, ^82^ ^]^ The ratio of the peak areas (see Table S2, Supporting Information) shows good agreement with literature IR data^[^ ^79^ ^]^ for the soluble BM3. The results suggest that based on protein secondary structure composition, the immobilized BM3 largely retains the native conformation of the enzyme in solution. The significant level of activity retention of the immobilized BM3 appears to be consistent with these findings.

## 4 Electrochemical Induced Nanoscale Stirring

In a next step, we asked whether the distinctive electrochemical properties of the MESA‐SAM on npAu are exploitable to tune the immobilization of BM3. In particular, we hypothesized that periodically altering the degree of ionization of the npAu/MESA through CVs, referred to here as “electrochemically induced nanoscale stirring”, could present a powerful means of enhancing specifically the dynamics of electrostatic interaction between the solid surface and BM3 fused to Z_basic2_.

Considering the requirement for the enzyme to penetrate fully into the pore network of the npAu/MESA, we adopted procedure from preliminary tests whereby 20 min breaks are inserted between every three cycles of voltammetry.

**Figure 5:**
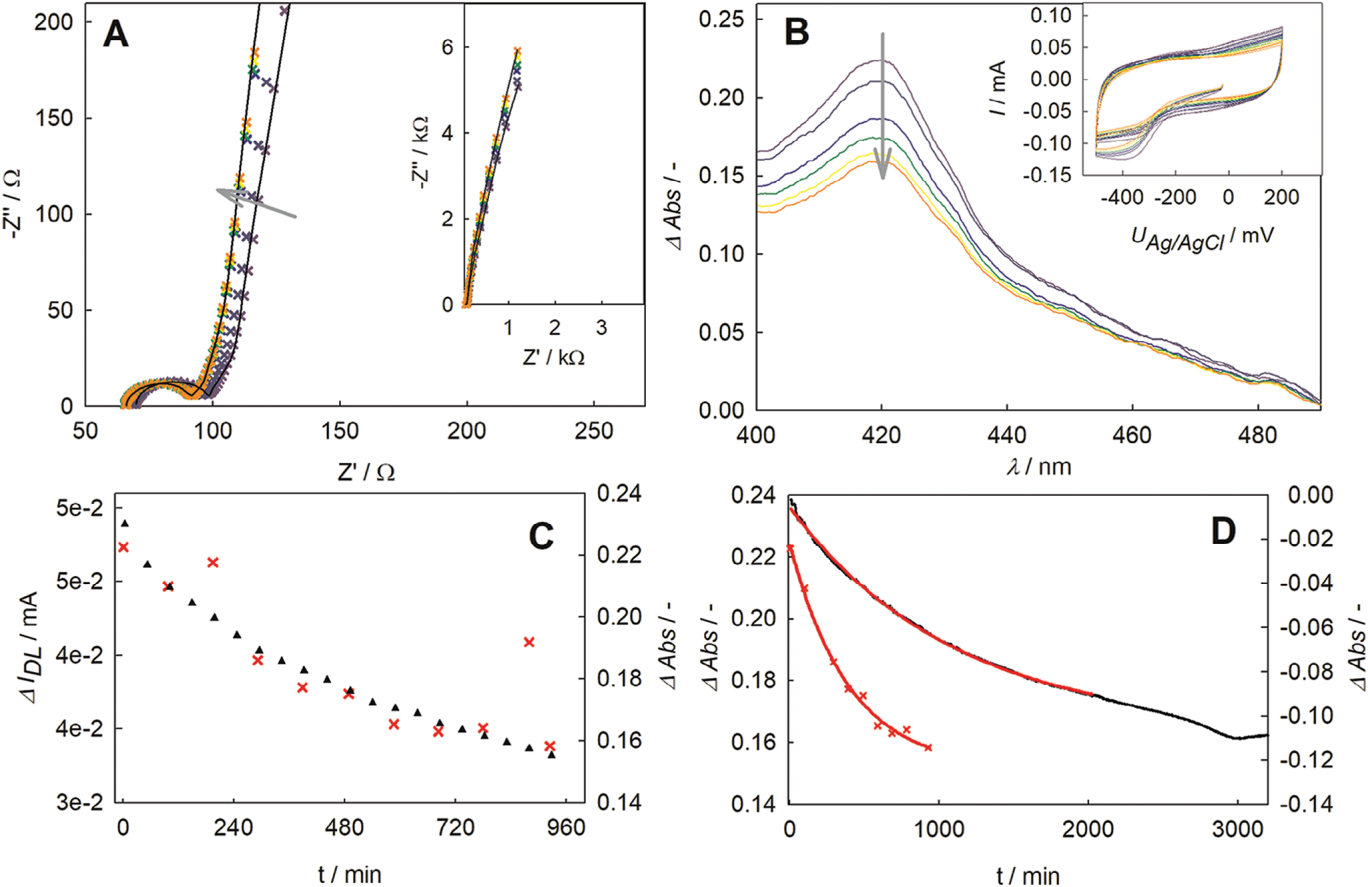
Electrochemically induced nanoscale stirring. A) Impedance spectra recorded in situ during the immobilization of BM3 on npAu/MESA. The inset shows the same data, plotted on different x‐ and y‐scales. B) Concomitant change in absorbance. The reference spectrum was recorded from buffer. Inset: CV done to induce the nanoscale stirring. Potential vs AgCl‐wire. For the sake of visibility, only the results for loops 1 (black), 3, 6, 10, 15, and 20 (orange) are plotted. The arrows indicate the shift of the spectra over time and same color coding was used for (A) and (B). C) Concomitant change of absorbance at 420 nm relative to 50 mm KPi (left axis; red stars) and change in the double layer current (black triangles, right axis) during CV as a function of incubation time. Double layer current was determined from the CVs in the inset in inset (B) at −100 mV after background correction. (Note: the two outliers in the UV/Vis dataset are presumably caused by background light in the laboratory as it is impossible to completely shield the cuvette.) D) Change of the absorbance at 420 nm replotted from (B) in red (stars) and from Figure S4 (Supporting Information) in black (solid line). The red curves represent the fit by an exponential decay.

To monitor the immobilization process, we analyzed the adsorption kinetics through a tailored combination of in situ EIS (**Figure** **5**A) and UV/Vis spectroscopy (Figure 5B). Comparing impedance spectra received from immobilization with and without nanoscale stirring (Figure 5A), we find that the change in the mid‐frequency regime is by far less pronounced when nanoscale stirring is used. The result implies a smaller degree of pore shape change under conditions of nanoscale stirring which is indicative of a more homogeneous distribution of the adsorbed proteins in the porous npAu/MESA structure. Moreover, the phase angle (low‐frequency regime) evolves stronger toward 90 °C (parallel to y‐axis) in the conditions of nanoscale stirring, implying a larger capacitance presumably due to denser packing of proteins. The decreasing relative shift between the individual spectra over time indicates saturation. Significantly, the reference immobilization without nanoscale stirring (see Figure 4A) did not show saturation during the same time range in the EIS data.

The UV/Vis data (Figure 5B) shows a decrease of 420 nm peak height, corresponding to the decrease of enzyme concentration in the supernatant. UV spectra of BM3 in solution (without npAu electrode) do not show any change over the same time period (Figure S8, Supporting Information). Consistent with the evolution of impedance spectra, the relative shift lessens over time. Absorption data monitoring the electrochemically enhanced as well as without nanoscale stirring immobilization are compared in Figure 5D. The process evolves 2.5‐fold faster upon electrochemical enhanced immobilization, which is revealed from the exponential decay fits. Temporal evolution of double layer current (from CVs in Figure 5B‐inset) and absorption peak decrease correlate very well, implying they result from the same kinetic process (Figure 5C). Relative to the reference immobilization without nanoscale stirring, the surface coverage reached was threefold higher when data on the depletion of enzyme concentration in supernatant are analyzed.

In conclusion, the presented strategy enables faster and more efficient immobilization, overcoming longstanding problems of surface accessibility of nanoporous electrodes. Thereby, multiple parameters play an important role. First, the scan rate during CV is directly linked to the frequency of surface charge variation. Here, it was set to 5 mVs^−1^ as trade‐off between increase in total measurement time (for lower scan rates) and too high background currents (for higher scan rates) which are potentially detrimental for the proteins. Yet, in the future, influence of scan rate variations could be interesting, in particular when applying other sample geometries. Secondly, the breaks between cycles are presumably beneficial for enzymes to penetrate the porous structure from the bulk solution (electrolyte) and the periods of electrochemical (potential) control to arrange and spread in the internal areas of the porous network. Thirdly, the surface charge distribution of BM3 (Figure 1B) most likely also contribute to the overall interaction between deprotonated (and partially protonated) SAM terminal groups and enzymes.

Lastly, rearrangement of the SAM^[^ ^83^ ^]^ as well as fluid motion in the pores^[^ ^84^ ^]^ induced by sweeping the electrode potential might also contribute to the observed behavior. Here, future studies could provide further in‐depth understanding of the system.

Studies in literature have already used variation/pulsing of the electrochemical potential for binding of biomolecules, e.g., potential‐pulses for enhanced DNA immobilization,^[^ ^85^, ^86^ ^]^ immobilization of [NiFe]‐hydrogenase on SAM (using titration to change the protonation state of the SAM terminal groups),^[^ ^87^ ^]^ adsorption of glucose oxidase on porous gold^[^ ^88^ ^]^ or adsorption of bilirubin oxidase on planar as well as npAu‐film electrodes (without SAMs) by applying periodic potential pulses below and above the point of zero charge (pzc).^[^ ^89^ ^]^ The important advance of the current approach is the facile exchange of the interfacial component parts as it primarily relies on the mutual interaction of the Z_basic2_ module and SAM terminal group.

## 5 Functionality of the npAu/MESA/BM3 Hybrid Electrode for Sensing Application

In terms of function, we considered the npAu/MESA/BM3 electrode primarily for mediated infusion of electrons into the BM3 active site for substrate hydroxylation. The mediator is NADP^+^/NADPH. Although well known in principle, electrochemical reduction of NAD(P)^+^ involves challenges of low efficiency and selectivity.^[^ ^40^, ^41^, ^90^ ^]^ Majority of studies are performed with NAD^+^/NADH, yet NADP^+^/NADPH regeneration was also shown on several electrode configurations.^[^ ^41^, ^91^, ^92^ ^]^ Bare metal electrodes were repeatedly discussed as electrocatalyst for NAD(P)H/NAD(P)^+^ regeneration,^[^ ^93^, ^94^, ^95^ ^]^ whereby nanostructured metal electrodes are particularly promising. Studies show that lower overpotentials are required by simultaneously achieving higher yields.^[^ ^92^, ^96^, ^97^, ^98^ ^]^ npAu was utilized for biosensor applications based on regeneration of NADH at significantly reduced overpotentials.^[^ ^99^, ^100^ ^]^

**Figure 6:**
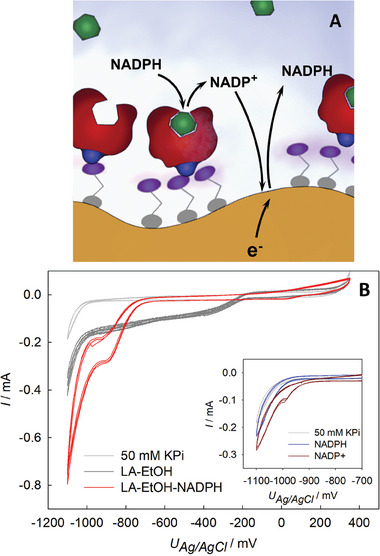
Electrochemical regeneration of NADPH using the npAu/MESA/BM3 electrode. A) Schematic representation of the reactive electrode surface. B) CVs of npAu/MESA/BM3 after reaction with 1.6 mm NADPH, and 1.6 mm lauric acid in 50 mm KPi (pH 7.5). For reference, CVs of npAu/MESA in 50 mm KPi (light grey) and npAu/MESA in 50 mm KPi containing 1.6 mm lauric acid (dark grey) are shown as well. Inset: CV of npAu/MESA in 50 mm KPi (pH 7.5) containing 1.6 mm NADP^+^ (dark red) and 1.6 mm NADPH (dark blue). All electrolytes were stirred magnetically (90 rpm). Total volume 4.5 mL.

For the present system, MESA assembled on npAu covers ≈70% of the surface (see Section 2 and Supplementary Methods). The electrochemically active surface area of the untreated npAu sample is 350 cm^2^ on average (determined via double layer charging as described in the Supplementary Information), implying that after surface modification with MESA and BM3 still ≈100 cm^2^ are available for regeneration of the coenzyme. In the complete electrode setup, NADPH is oxidized to NADP^+^ during the enzymatic reaction. The electrode is then expected to provide pristine gold in sufficient degree to enable direct electrochemical interaction with NADP^+^ for subsequent reduction to NADPH, as depicted in **Figure** **6**A.

First, electrochemistry of NADP^+^/NADPH containing electrolytes are investigated. CVs of npAu/MESA in NADP^+^ indeed exhibit a clear reduction peak (Figure 6B inset). Reference measurements did not show peaks in the same potential range, hence interfering redox‐reactions^[^ ^90^ ^]^ are excluded. The peak characteristics are qualitatively well in line with NAD(P)^+^ reduction reported in literature^[^ ^94^ ^]^ and thus indicate a successful conversion of NADP^+^.

In the next step, we investigated electrochemical reduction of the NADP^+^ released by enzymatic reaction of the npAu/MESA/BM3 electrode (Figure 6B). Voltammograms recorded after 2 h (Figure 6B) identify the reduction of NADP^+^. As immobilized BM3 was used, the NADP^+^ is directly formed in the porous structure which explains the strong peak. We note a slight current shift in the CVs (Figure 6B – dark grey line) due to change in electrolyte composition resulting from ethanol co‐solvent (2%) used for the solubilization of lauric acid.

Figure 6B shows the observed reduction peak at −900 mV. Peak potential of −1.14 V was reported for NAD^+^ reduction on polycrystalline gold.^[^ ^94^ ^]^ Assuming reduction of NAD^+^ and NADP^+^ to occur at the same potential as supported by studies on glassy carbon electrodes,^[^ ^101^ ^]^ nanoporous gold decreases the necessary overpotential by roughly 240 mV. This can be correlated to the intrinsic catalytic properties of nanoporous surfaces.^[^ ^19^ ^]^ Stability of the SAM during these electrochemical procedures was verified by subsequent analysis of oxidative desorption in 1 m KOH (see Figure S9, Supporting Information). Production of unwanted by‐products (e.g., dimerization) is one of the major constraints when applying electrochemical regeneration of NAD(P)H.^[^ ^90^, ^91^ ^]^ Although further investigation of the electrochemical reaction goes beyond the scope of the present study, we analyzed the usage of electrochemically regenerated NADPH during the biocatalytic hydroxylation of lauric acid by BM3. The reaction was set up with NADP^+^, lauric acid, soluble BM3 (0.45 µm), and npAu/MESA electrode. A potential of ‐1040 mV, hence more negative than the reduction peak potential in Figure 6B, was applied (data not shown). Soluble BM3 was chosen here on purpose due to the long timespans needed for mixing and equilibration of the system. The reaction mixture was analyzed by GC‐FIC (Figure S10, Supporting Information) and the release of 40 µm hydroxylauric acid after 3.5 h confirms the electrochemical reduction of NADP^+^, with the resulting NADPH productively used by the BM3 reaction in solution. Overoxidation of substrate (i.e., formation of doubly hydroxylated lauric acid; see Buergler et al.^[^ ^44^ ^]^) was not observed. The same experiment was conducted at a potential (−850 mV) more positive than the reduction peak of NADP^+^ (data not shown) and hydroxylauric acid was not formed, as expected. For reference, we supplied NADPH directly to the reaction of npAu/MESA/BM3 and find that the rate of release of hydroxylauric acid (43 µm in 2 h) was roughly twice that of the experiment involving electrochemically generated NADPH and enzymatic reaction in solution. As the immobilized BM3 was present only at half the concentration of the soluble enzyme, the difference in rate normalized on enzyme used is therefore fourfold or higher. These comparisons identify the electrochemical reduction of NADP^+^ as a prime rate‐limiting factor of the reaction with the soluble BM3 and suggest optimization of its kinetics as a relevant target of future research.

Lastly, we also examined direct electron transfer (DET) from the electrode to BM3. CVs of npAu/MESA/BM3 were recorded in electrolyte containing lauric acid but without NADP^+^. The O_2_ concentration in solution was also measured simultaneously. If electron transfer occurred, an increased cathodic current (reduction) typical for a catalytically active system should be detected.^[^ ^2^ ^]^ However, relative to npAu/MESA reference without BM3, no additional electron transfer was observed (shown in Figure S11, Supporting Information). Moreover, no O_2_ was consumed (Figure S11, Supporting Information – inset). Essential for DET is the electron tunneling distance between the surface and the enzyme active site which according to literature must not exceed 1.5‐2.0 nm.^[^ ^9^ ^]^ Based on the PDB structure for the P450 domain (PDB: 4kew), the distance between the N‐terminus and heme (active site) is 3.6 nm. This explains why DET could not be observed for the present system of npAu/MESA/BM3 (fused to Z_basic2_ module). However, in that perspective applying structurally different P450s (or other enzyme classes) in the same electrode setup might be interesting for further study.

## 6 Conclusion

Based on study of BM3 on npAu, we here present a new and modular approach of enzyme electrode interface design. Charge‐directed enzyme immobilization is achieved via the cationic binding module Z_basic2_ that is fused to BM3. An anionic SAM on the npAu surface attracts the adsorption of Z_basic2_. The electrode‐adsorbed BM3 retains well the activity (33%) and selectivity (complete) from in‐solution. By inducing a nanoscale stirring effect in the rigid channel network, the speed of enzyme adsorption and the surface density of immobilized enzyme could be increased substantially (≥2.5‐fold). We expect the strategy conceptualized here to be applicable broadly, and hence to be of general significance, for P450 enzymes and various other oxidoreductases. Finally, electrochemical regeneration of NADPH was achieved. The strongly decreased overpotential (Δ = 240 mV) necessary for the reduction of NADP^+^ is correlated to the structure‐related catalytic activity of the nanoporous structure. Collectively, our findings make a strong case for the use of electrochemistry to enhance electrostatic immobilization of Z_basic2_‐enzyme fusions on npAu. They further showcase the application of npAu as electrode material for functional enzymatic biosensors.

## 7 Experimental Section

### Materials

All chemicals were from Sigma‐Aldrich or Carl Roth. For electrolyte preparation, 1 m perchloric acid (HClO_4_, > 99%), potassium hydroxide (KOH, ≥85%), potassium chloride, monopotassium phosphate (KH_2_PO_4_, ≥99%), and dipotassium phosphate (K_2_HPO_4_ ≥99%) were dissolved or diluted, respectively, to the desired concentration in high purity water (ROTIPURAN or Milli‐Q). Potassium phosphate buffer (KPi) was prepared from KH_2_PO_4_ and K_2_HPO_4_.

### Preparation of npAu/MESA/BM3 Electrodes

*npAu*: AuAg (25/75 at.%) was prepared by arc melting and afterward homogenized under argon atmosphere at 800 °C for 12 h. The alloy was then rolled to a thickness of ≈150 µm and annealed at 600 °C for 1 h in a vacuum oven (10^−6^ mbar). Finally, it was cut into small pieces (platelets) in the size of ≈5 × 5 mm (80 mg). Electrical contact was achieved by wrapping the samples with a gold wire (0.25 mm diameter) and subsequent pressing with a conventional hand press. Electrochemical dealloying was done in 0.1 m HClO_4_ at +1100 mV until current dropped below −5 µA. Samples were thermally annealed for 2 h at varying temperatures between 70 and 400 °C in a vacuum oven (10^−6^ mbar). After rinsing with distilled water, the samples were cycled in fresh 0.1 m HClO_4_ to remove any oxide layer formed during etching (−200 to +1200 mV, 0.5 mVs^−1^, 2 cycles). The final pore size was determined electrochemically by recording CVs in the double layer regime at varying scan rates between 20 and 45 mVs^−1^ (in 0.1 m HClO_4_), as explained in the Supplementary Information (see also Figure S1, Supporting Information). Samples were thoroughly rinsed with water before further use. Prior to surface modifications, CVs were recorded in 1 m KOH for activation of the surface (+600 to ‐1000 mV, 2 mVs^−1^, 2–3 cycles).

*npAu/MESA*: 2‐Mercaptoethanesulfonate (sodium salt; HSCH_2_CH_2_SO_3_Na, MESA, 98%) was used for surface modification. The npAu‐samples were immersed in 5 mL of 5 mm solution for 48 h. The beaker was sealed with parafilm and wrapped in aluminum foil for UV‐shielding. After incubation, the sample was immersed in water for 1 h and used immediately for the respective measurements to exclude any influences from storage.

*Enzyme Preparation*: Production and purification of BM3 were done following Valikhani et al.^[^ ^26^ ^]^ as described in the Supporting Information. Specific activity of purified BM3 was 0.6 U/mg. One unit (U) equals 1 µmol of O_2_ converted per minute during the hydroxylation of lauric acid. Enzyme concentration was quantified via CO titration,^[^ ^102^, ^103^ ^]^ for details see Supporting Information.

*Enzyme Immobilization*: Before incubation in the enzyme solution, the npAu/MESA samples (≈30 mg each) were first rinsed with water and equilibrated in 50 mm KPi for 1 h. The specific absorption peak of P450s at 420 nm was used to monitor the concentration decrease during immobilization of BM3 on npAu/MESA by UV/Vis spectroscopy. Two cuvettes were mounted in a spectrometer, one including a npAu/MESA sample mounted well above the optical path and the other containing only the enzyme solution (0.6 µm BM3 in 50 mm KPi, pH 7.5). The absorption at 420 nm was then monitored over time. The immobilization kinetics was analyzed thus.

Standard immobilizations were done by immersing the npAu sample in 800 µL of 120 ± 50 µg mL^−1^ (1.0 ± 0.4 µm). The conditions used were as follows: 50 mm KPi, pH 7.5, incubation time 15 h, 4 °C. Afterward, the samples were rinsed by immersing them in 50 mm KPi, pH 7.5, for 1 h. The immobilization yield is the percentage of bound enzyme compared to total amount of offered enzyme in the supernatant and is calculated according to (c_0_ − c_s_)/c_0_ × 100, with c_0_ the initial concentration, and c_s_ the concentration in the supernatant after immobilization was performed.

### Electrochemical Measurements

If not stated otherwise, all electrochemical measurements were performed in a beaker (15‐20 mL) at room temperature (∼22 °C) and ambient air using a three‐electrode setup and controlled by an Autolab PGSTAT204 or PGSTAT128N instrument, equipped with a FRA module and a spectrophotometer unit, from Metrohm.

The reference electrode (RE) is a commercial Ag/AgCl, filled with saturated KCl or, in case of electrochemical dealloying, containing a KCl‐KNO_3_ salt bridge. All potentials are given versus Ag/AgCl. A self‐made AgCl‐wire substituted the commercial RE if measurement was done at 4 °C or the total reaction volume did not allow usage of full‐size electrodes. To prepare the AgCl‐wire, Ag‐wires were immersed in 1 m KCl solution, and current density of +1 mA cm^−2^ was applied for 3 min. To obtain a denser coating, the current was reversed every 30 s for 5 s. Applied potential was adjusted individually versus AgCl‐wire relative to the commercial RE.

As counter electrode (CE), a curled Pt‐wire (solely for electrochemical dealloying) or a carbon cloth was used. If assembled in an Eppendorf tube or cuvette, an Au‐wire was used instead.

For EIS, frequencies between 100 kHz and 1mHz with a peak amplitude of 10 mV were used and a curled Pt‐wire and AgCl‐wire served as RE and CE, respectively. Hereby, the cell was mounted in an Eppendorf tube (0.8 mL) or semi‐micro cuvette (1.3 mL) if accompanied by UV/Vis spectroscopy. EEC fitting was done with the impedance data analysis tool of the NOVA software by Metrohm.

Electrochemical nanoscale stirring was done via CVs between −500 and +200 mV (vs AgCl‐wire) at a scan rate of 5 mVs^−1^ (three cycles) followed by a 20 min break (constant potential) and in situ EIS. This procedure was repeated for 20 loops resulting in a total experiment time of 15.5 h (at 4 °C). UV/Vis spectra were recorded after each loop.

### In Situ Resistometry

Electrochemical analysis of the MESA‐layer on npAu was done using in situ four‐point resistance measurements and concomitant CVs according to the previous work (Hengge et al.^[^ ^55^ ^]^) and is shortly summarized in the Supplementary Information.

### Biochemical Analysis

*Enzyme Activity*: To quantify the activity of soluble as well as immobilized enzyme, the oxygen consumption during the hydroxylation of lauric acid was monitored. This was achieved by inserting an optical oxygen sensor (OXROB10, Pyroscience GmbH, Aachen, Germany coupled to a FireStingO2 (FSO2‐x) fiber‐optic oxygen meter) in the reaction volume. Lauric acid (C_12_H_24_O_2_) was used as the substrate with the following reaction conditions (if not stated otherwise): 200 µm lauric acid, 200 µm NAPDH in 50 mm KPi buffer, pH 7.5, at 30 °C, magnetic stirred at 300 rpm. Depending on the desired measurements, either 200 µL of enzyme solution or the npAu electrode was added to start the reaction. The total volume of 2 mL. The enzyme effectiveness is the ratio of the observed activity of the immobilized enzyme to the activity of the same amount of soluble enzyme, i.e., the theoretical activity of the immobilized enzyme.

*Product Formation*: The release of hydroxylauric acid was measured. Gas chromatography (GC) was done following Valikhani et al.^[^ ^26^ ^]^ with modification adopted from Buergler et al.^[^ ^44^ ^]^ GC chromatograms shown in Figures S4 and S7 (Supporting Information) were obtained using a Shimadzu GC‐2010 with a Zebron‐5MS column (He carrier gas) with temperature program as follows: start at 100 °C; hold for 5 min; rise at 10 °C min^−1^ to 300 °C, hold 0 min. The injection temperature was 300 °C. Calibration for quantitative analysis is shown in Figure S12 (Supporting Information).

### Complementary Characterization Methods

*SEM*: To prepare sample for scanning electron microscopy (SEM), one npAu sample (10 × 5 mm, instead of 5 × 5 mm) was cut in half after MESA surface modification. One part was subsequently used for enzyme immobilization. The other half served as the reference sample. Parameters for surface modification and enzyme immobilization were identical to all other samples. After immobilization, the npAu sample was rinsed with buffer solution and then freeze dried. Each sample was subsequently halved and placed upright (see Figure S7, Supporting Information) on the sample holder in the SEM (Zeiss Ultra 55) for analysis. A high voltage of 1.0 kV and a spot size of 10 was used.

*GIR‐FTIR*: A gold infrared mirror served as carrier material for grazing incidence reflectance Fourier Transform Infrared spectroscopy (GIR‐FTIR). One was surface modified with MESA and a second sample with MESA and BM3, respectively. For all preparation steps, the same parameters were used as for the nanoporous samples. The samples were rinsed with buffer and distilled water. The spectra were recorded by a FT‐IR Microscope Bruker Tensor 27 with Hyperion 3000 and a single‐element‐MCT detector and a GIR objective. Data analysis including self‐deconvolution and fitting was done with OriginPro2022.

## Conflict of Interest

The authors declare no conflict of interest.

## Supporting information



Supporting Information

## Data Availability

The data that support the findings of this study are available from the corresponding author upon reasonable request.

## References

[1] D. G. Boucher , E. Carroll , Z. A. Nguyen , R. G. Jadhav , O. Simoska , K. Beaver , S. D. Minteer , Angew. Chem., Int. Ed. 2023, 62, e202307780. 10.1002/anie.202307780 37428529

[2] I. Mazurenko , V. P. Hitaishi , E. Lojou , Curr. Opin. Electrochem. 2020, 19, 113.

[3] A. Ruff , F. Conzuelo , W. Schuhmann , Nat. Catal. 2020, 3, 214.

[4] N. J. Ronkainen , H. B. Halsall , W. R. Heineman , Chem. Soc. Rev. 2010, 39, 1747. 20419217 10.1039/b714449k

[5] F. Otero , E. Magner , Sensors 2020, 20, 3561. 32586032 10.3390/s20123561 PMC7349852

[6] X. Xiao , P. Si , E. Magner , Bioelectrochemistry 2016, 109, 117. 26781363 10.1016/j.bioelechem.2015.12.008

[7] K. J. Stine , Biochem. Insights 2017, 10, 117862641774860. 10.1177/1178626417748607 PMC5751899 29308011

[8] P. Sondhi , D. Lingden , J. K. Bhattarai , A. V. Demchenko , K. J. Stine , Metals 2023, 13, 78. 39238564 10.3390/met13010078 PMC11376205

[9] X. Yan , J. Tang , D. Tanner , J. Ulstrup , X. Xiao , Catalysts 2020, 10, 1458.

[10] T. Siepenkoetter , U. Salaj‐Kosla , E. Magner , ChemElectroChem 2017, 4, 905.

[11] H. Qiu , L. Xue , G. Ji , G. Zhou , X. Huang , Y. Qu , P. Gao , Biosens. Bioelectron. 2009, 24, 30143018. 10.1016/j.bios.2009.03.011 19345571

[12] C. Wu , X. Liu , Y. Li , X. Du , X. Wang , P. Xu , Biosens. Bioelectron. 2014, 53, 2630. 10.1016/j.bios.2013.09.040 24121205

[13] T. Siepenkoetter , U. Salaj‐Kosla , X. Xiao , P. Ó Conghaile , M. Pita , R. Ludwig , E. Magner , ChemPlusChem 2016, 82, 553560. 10.1002/cplu.201600455 31961582

[14] U. Salaj‐Kosla , S. Pöller , Y. Beyl , M. D. Scanlon , S. Beloshapkin , S. Shleev , W. Schuhmann , E. Magner , Electrochem. Commun. 2012, 16, 9295.

[15] N. J. Francis , C. R. Knospe , Adv. Eng. Mater. 2019, 21, 1800857.

[16] J. Erlebacher , I. McCue , Acta Mater. 2012, 60, 6164.

[17] E. Seker , M. Reed , M. Begley , Materials 2009, 2, 21882215.

[18] J. Weissmüller , R. C. Newman , H.‐J. Jin , A. M. Hodge , J. W. Kysar , MRS Bull. 2009, 34, 577.

[19] G. Wittstock , M. Bäumer , W. Dononelli , T. Klüner , L. Lührs , C. Mahr , L. V. Moskaleva , M. Oezaslan , T. Risse , A. Rosenauer , A. Staubitz , J. Weissmüller , A. Wittstock , Chem. Rev. 2023, 123, 6716. 37133401 10.1021/acs.chemrev.2c00751 PMC10214458

[20] E.‐M. Steyskal , M. Seidl , M. Graf , R. Würschum , Phys. Chem. Chem. Phys. 2017, 19, 29880. 29086785 10.1039/c7cp05706g

[21] M. Gößler , M. Albu , G. Klinser , E.‐M. Steyskal , H. Krenn , R. Würschum , Small 2019, 15, 1904523. 10.1002/smll.201904523 31573141

[22] E. Hengge , M. Hirber , P. Brunner , E.‐M. Steyskal , B. Nidetzky , R. Würschum , Phys. Chem. Chem. Phys. 2021, 23, 14457. 34184015 10.1039/d1cp01491a

[23] T. Nöll , G. Nöll , Chem. Soc. Rev. 2011, 40, 3564. 21509355 10.1039/c1cs15030h

[24] J. C. Love , L. A. Estroff , J. K. Kriebel , R. G. Nuzzo , G. M. Whitesides , Chem. Rev. 2005, 105, 1103. 15826011 10.1021/cr0300789

[25] C. Vericat , M. E. Vela , G. Benitez , P. Carro , R. C. Salvarezza , Chem. Soc. Rev. 2010, 39, 1805. 20419220 10.1039/b907301a

[26] D. Valikhani , J. M. Bolivar , A. Dennig , B. Nidetzky , Biotechnol. Bioeng. 2018, 115, 2416. 30036448 10.1002/bit.26802 PMC6836874

[27] J. M. Bolivar , B. Nidetzky , Langmuir 2012, 28, 10040. 22668007 10.1021/la3012348

[28] J. M. Bolivar , V. Gascon , C. Marquez‐Alvarez , R. M. Blanco , B. Nidetzky , Langmuir 2017, 33, 5065. 28464607 10.1021/acs.langmuir.7b00441

[29] P. Wied , F. Carraro , J. M. Bolivar , C. J. Doonan , P. Falcaro , B. Nidetzky , Angew. Chem., Int. Ed. 2022, 61, e202117345. 10.1002/anie.202117345 PMC9305891 35038217

[30] T. Gräslund , G. Lundin , M. Uhlén , P.‐Å. Nygren , S. Hober , Protein Eng., Des. Sel. 2000, 13, 703. 10.1093/protein/13.10.703 11112509

[31] J. M. Bolivar , B. Nidetzky , Biotechnol. Bioeng. 2012, 109, 1490. 22249953 10.1002/bit.24423

[32] A. W. Munro , D. G. Leys , K. J. McLean , K. R. Marshall , T. W. B. Ost , S. Daff , C. S. Miles , S. K. Chapman , D. A. Lysek , C. C. Moser , C. C. Page , P. L. Dutton , Trends Biochem. Sci. 2002, 27, 250. 12076537 10.1016/s0968-0004(02)02086-8

[33] Q. Dai , L. Yang , Y. Wang , X. Cao , C. Yao , X. Xu , Anal. Bioanal. Chem. 2020, 412, 4703. 32483647 10.1007/s00216-020-02727-0

[34] V. E. V. Ferrero , L. Andolfi , G. Di Nardo , S. J. Sadeghi , A. Fantuzzi , S. Cannistraro , G. Gilardi , Anal. Chem. 2008, 80, 8438. 18947200 10.1021/ac8011413

[35] P. Panicco , Y. Astuti , A. Fantuzzi , J. R. Durrant , G. Gilardi , J. Phys. Chem. B 2008, 112, 14063. 18842012 10.1021/jp8050033

[36] A. K. Udit , K. D. Hagen , P. J. Goldman , A. Star , J. M. Gillan , H. B. Gray , M. G. Hill , J. Am. Chem. Soc. 2006, 128, 10320. 16881664 10.1021/ja061896w

[37] A. Pardo‐Jacques , R. Basseguy , A. Bergel , Electrochim. Acta 2006, 52, 979.

[38] B. D. Fleming , Y. Tian , S. G. Bell , L.‐L. Wong , V. Urlacher , H. A. O. Hill , Eur. J. Biochem. 2003, 270, 4082. 14519119 10.1046/j.1432-1033.2003.03799.x

[39] D. Valikhani , J. M. Bolivar , J. N. Pelletier , ACS Catal. 2021, 11, 9418.

[40] H. Wu , C. Tian , X. Song , C. Liu , D. Yang , Z. Jiang , Green Chem. 2013, 15, 1773.

[41] Y. S. Lee , R. Gerulskis , S. D. Minteer , Curr. Opin. Biotechnol. 2022, 73, 14. 34246871 10.1016/j.copbio.2021.06.013

[42] S. Kochius , A. O. Magnusson , F. Hollmann , J. Schrader , D. Holtmann , Appl. Microbiol. Biotechnol. 2012, 93, 2251. 22327354 10.1007/s00253-012-3900-z

[43] E. Jurrus , D. Engel , K. Star , K. Monson , J. Brandi , L. E. Felberg , D. H. Brookes , L. Wilson , J. Chen , K. Liles , M. Chun , P. Li , D. W. Gohara , T. Dolinsky , R. Konecny , D. R. Koes , J. E. Nielsen , T. Head‐Gordon , W. Geng , R. Krasny , G.‐W. Wei , M. J. Holst , J. A. McCammon , N. A. Baker , Protein Sci. 2018, 27, 112. 28836357 10.1002/pro.3280 PMC5734301

[44] M. B. Buergler , A. Dennig , B. Nidetzky , Biotechnol. Bioeng. 2020, 117, 2377. 32369187 10.1002/bit.27372 PMC7384007

[45] Y. Ding , Y.‐J. Kim , J. Erlebacher , Adv. Mater. 2004, 16, 1897.

[46] F. Kertis , J. Snyder , L. Govada , S. Khurshid , N. Chayen , J. Erlebacher , JOM 2010, 62, 50.

[47] Y. Sun , X. Huang , Y. Osawa , Y. E. Chen , H. Zhang , Molecules 2023, 28, 5353. 37513226 10.3390/molecules28145353 PMC10383305

[48] P. Urban , D. Pompon , Sci. Rep. 2022, 12, 15982. 36155638 10.1038/s41598-022-20390-6 PMC9510131

[49] H. Zhang , A. L. Yokom , S. Cheng , M. Su , P. F. Hollenberg , D. R. Southworth , Y. Osawa , J. Biol. Chem. 2018, 293, 7727. 29618513 10.1074/jbc.RA117.000600 PMC5961045

[50] E. Detsi , E. Jong , A. Zinchenko , Z. Vuković , I. Vuković , S. Punzhin , K. Loos , G. Brinke , H. A. Raedt , P. R. Onck , J. T. M. Hosson , Acta Mater. 2011, 59, 7488.

[51] C. Lakshmanan , R. Viswanath , S. Polaki , R. Rajaraman , AIP Conf. Proc. 2015, 1665, 140033.

[52] J. Wang , R. Xia , J. Zhu , Y. Ding , X. Zhang , Y. Chen , J. Mater. Sci. 2012, 47, 5013.

[53] J. Erlebacher , Phys. Rev. Lett. 2011, 106, 225504. 21702615 10.1103/PhysRevLett.106.225504

[54] C. M. Winkeljohn , S. Shahriar , E. Seker , J. K. Mason , Comput. Mater. Sci. 2023, 230, 112430.

[55] E. Hengge , E.‐M. Steyskal , R. Bachler , A. Dennig , B. Nidetzky , R. Würschum , Beilstein J. Nanotechnol. 2019, 10, 2275. 31807412 10.3762/bjnano.10.219 PMC6880825

[56] C. M. Fox , C. B. Breslin , J. Appl. Electrochem. 2020, 50, 125.

[57] P. Laibinis , M. A. Fox , J. P. Folkers , G. Whitesides , Langmuir 1991, 7, 3167.

[58] A. Stewart , S. Zheng , M. R. McCourt , S. E. J. Bell , ACS Nano 2012, 6, 3718. 22500816 10.1021/nn300629z PMC3614020

[59] M. Smiljanić , C. Adam , T. Doneux , J. Electroanal. Chem. 2018, 815, 238.

[60] C. P. Smith , H. S. White , Langmuir 1993, 9, 13.

[61] I. Burgess , B. Seivewright , R. B. Lennox , Langmuir 2006, 22, 4420. 16618197 10.1021/la052767g

[62] A. M. Luque , W. H. Mulder , J. J. Calvente , A. Cuesta , R. Andreu , Anal. Chem. 2012, 84, 5778. 22668082 10.1021/ac301040r

[63] W. R. Fawcett , M. Fedurco , Z. Kovacova , Langmuir 1994, 10, 2403.

[64] J.‐J. Shyue , M. R. Guire , T. Nakanishi , Y. Masuda , K. Koumoto , C. N. Sukenik , Langmuir 2004, 20, 8693. 15379494 10.1021/la049247q

[65] J. F. Smalley , K. Chalfant , S. W. Feldberg , T. M. Nahir , E. F. Bowden , J. Phys. Chem. B 1999, 103, 1676.

[66] Z. Dai , H. Ju , Phys. Chem. Chem. Phys. 2001, 3, 3769.

[67] T. Kakiuchi , M. Iida , S. Imabayashi , K. Niki , Langmuir 2000, 16, 5397.

[68] R. Goseki , T. Ishizone , Encyclopedia of Polymeric Nanomaterials, Springer, Berlin, Heidelberg, Germany 2015.

[69] F. Habashi , Encyclopedia of Metalloproteins, Springer, New York, USA 2013.

[70] S. Meng , Y. Ji , L. Zhu , G. V. Dhoke , M. D. Davari , U. Schwaneberg , Biotechnol. Adv. 2022, 61, 108051. 36270499 10.1016/j.biotechadv.2022.108051

[71] M. A. MacDonald , H. A. Andreas , Electrochim. Acta 2014, 129, 290.

[72] R. de Levie , Electrochim. Acta 1963, 8, 751.

[73] H. Keiser , K. D. Beccu , M. A. Gutjahr , Electrochim. Acta 1976, 21, 539.

[74] Q. Xie , C. Xiang , Y. Yuan , Y. Zhang , L. Nie , S. Yao , J. Colloid Interface Sci. 2003, 262, 107. 16256587 10.1016/S0021-9797(03)00196-6

[75] S. D. Keighley , P. Li , P. Estrela , P. Migliorato , Biosens. Bioelectron. 2008, 23, 1291. 18178423 10.1016/j.bios.2007.11.012

[76] M. Wang , L. Wang , G. Wang , X. Ji , Y. Bai , T. Li , S. Gong , J. Li , Biosens. Bioelectron. 2004, 19, 575. 14683641 10.1016/s0956-5663(03)00252-5

[77] X. Wang , X. Liu , X. Yan , P. Zhao , Y. Ding , P. Xu , J. R. Lu , PLoS One 2011, 6, 24207. 10.1371/journal.pone.0024207 PMC3166289 21912676

[78] K. K. Chittur , Biomaterials 1998, 19, 357. 9677150 10.1016/s0142-9612(97)00223-8

[79] C. Jung , Anal. Bioanal. Chem. 2008, 392, 1031. 18581103 10.1007/s00216-008-2216-4

[80] B.‐J. Niu , M. W. Urban , J. Appl. Polym. Sci. 1996, 62, 1903.

[81] J. Kong , S. Yu , Acta Biochim. Biophys. Sin. 2007, 39, 549. 17687489 10.1111/j.1745-7270.2007.00320.x

[82] A. Sadat , I. J. Joye , Appl. Sci. 2020, 10, 5918.

[83] J. Zhang , A. Demetriou , A. C. Welinder , T. Albrecht , R. J. Nichols , J. Ulstrup , Chem. Phys. 2005, 319, 210.

[84] Y. Xue , J. Markmann , H. Duan , J. Weissmller , P. Huber , Nat. Commun. 2014, 5, 1. 10.1038/ncomms5237 PMC4102117 24980062

[85] F. Fixe , R. Cabeça , V. Chu , D. M. F. Prazeres , G. N. M. Ferreira , J. P. Conde , Appl. Phys. Lett. 2003, 83, 1465.

[86] D. Jambrec , M. Gebala , F. L. Mantia , W. Schuhmann , Angew. Chem., Int. Ed. 2015, 54, 15064. 10.1002/anie.201506672 26487262

[87] Y. Wang , Z. Kang , L. Zhang , Z. Zhu , ACS Catal. 2022, 12, 1415.

[88] H. du Toit , M. Di Lorenzo , Electrochim. Acta 2014, 138, 86.

[89] F. Lopez , T. Siepenkoetter , X. Xiao , E. Magner , W. Schuhmann , U. Salaj‐Kosla , J. Electroanal. Chem. 2018, 812, 194.

[90] E. Aamer , J. Thöming , M. Baune , N. Reimer , R. Dringen , M. Romero , I. Bösing , Sci. Rep. 2022, 12, 16380. 36180530 10.1038/s41598-022-20508-w PMC9525651

[91] Y. Li , G. Liu , W. Kong , S. Zhang , Y. Bao , H. Zhao , L. Wang , L. Zhou , Y. Jiang , Green Chem. Eng. 2024, 5, 1.

[92] J. T. Kadowaki , T. H. Jones , A. Sengupta , V. Gopalan , V. V. Subramaniam , Sci. Rep. 2021, 11, 180. 33420179 10.1038/s41598-020-79761-6 PMC7794519

[93] H. Jaegfeldt , Bioelectrochem. Bioenerg. 1981, 8, 355.

[94] A. Damian , S. Omanovic , J. Mol. Catal. A: Chem. 2006, 253, 222.

[95] I. Ali , T. Khan , S. Omanovic , J. Mol. Catal. A: Chem. 2014, 387, 86.

[96] I. Ali , A. Gill , S. Omanovic , J. Chem. Eng. 2012, 188, 173.

[97] R. Barin , S. Rashid‐Nadimi , D. Biria , M. A. Asadollahi , Electrochim. Acta 2017, 247, 1095.

[98] J. W. H. Burnett , H. Chen , J. Li , Y. Li , S. Huang , J. Shi , A. J. McCue , R. F. Howe , S. D. Minteer , X. Wang , ACS Appl. Mater. Interfaces 2022, 14, 20943. 35482431 10.1021/acsami.2c01743

[99] Y. Mie , Y. Yasutake , M. Ikegami , T. Tamura , Sens. Actuators, B 2019, 288, 512.

[100] F. Otero , T. Mandal , D. Leech , E. Magner , Sens. Actuators Rep. 2022, 4, 100117.

[101] K. Vuorilehto , S. Lütz , C. Wandrey , Bioelectrochemistry 2004, 65, 1. 15522685 10.1016/j.bioelechem.2004.05.006

[102] T. Omura , R. Sato , J. Biol. Chem. 1964, 239, 2379. 14209972

[103] A. Dennig , N. Lülsdorf , H. Liu , U. Schwaneberg , Angew. Chem., Int. Ed. 2013, 52, 8459. 10.1002/anie.201303986 23818430

