# Supplementary material for: Electrochemically Induced Nanoscale Stirring Boosts Functional Immobilization of Flavocytochrome P450 BM3 on Nanoporous Gold Electrodes
Source: Small Methods. 2024 Sep 19;9(3):2400844. doi: 10.1002/smtd.202400844 (PMC11926518; doi:10.1002/smtd.202400844)
Supplement: Supplementary file 1 — Supporting Information [file SMTD-9-2400844-s001.docx]

Supporting Information

Electrochemically induced nanoscale stirring boosts functional immobilization of flavocytochrome P450 BM3 on nanoporous gold electrodes

Elisabeth Hengge, Eva-Maria Steyskal, Alexander Dennig, Manfred Nachtnebel, Harald Fitzek, Roland Würschum and Bernd Nidetzky*

E. Hengge, A. Dennig, B. Nidetzky

Institute of Biotechnology and Biochemical Engineering, Graz University of Technology, Petersgasse 12, 8010 Graz, Austria
E-mail: bernd.nidetzky@tugraz.at

B. Nidetzky
Austrian Centre of Industrial Biotechnology (acib), Petersgasse 14, 8010 Graz, Austria

E. Hengge, E.-M. Steyskal, R. Würschum
Institute of Materials Physics, Graz University of Technology, Petergasse 16, 8010 Graz, Austria

M. Nachtnebel, H. Fitzek
Graz Centre for Electron Microscopy (ZFE), Steyrergasse 17, 8010 Graz, Austria

**Supporting Methods**

Electrochemical pore size determination

Pore size analysis was conducted following Detsi et al.^1^ and Lakshmanan et al ^2^. Cyclic voltammograms were recorded in the double layer regime at scan rates between 20 and 45 mVs^-1^ (Figure S1(a)). The linear fit of the current *versus* scan rate (Figure S1(b)) gives the total capacitance of the sample (slope *a*) and the pore size *d* can be estimated, according to

$$d=\frac{mk}{\rho a}*C_{spec}$$

with *m* the mass in gram, ρ the density of gold^3^ (19.3 g/cm^3^) , *C_spec_* the specific interfacial capacitance^2^ (40 µF*/*cm^2^) and *k* an empirical dimensionless parameter (*k* = 3*.*7 for disordered nanoporous structures^1^).

From these measurement, the total active surface area (*A*) can be determined via $A=\frac{C_{spec}}{a}$ .

In situ resistometry

One npAu sample with 90 nm pore size was connected with 5 wires, 4 to enable four-point resistometry and the middle one for electrochemical control. The sample was first equilibrated in 10 mL of high purity water for 1.5 h. Then 10 mL of 10 mM MESA (final concentration 5mM) was added. The evolution of relative change in resistance is shown in Figure S3A. The process takes at least 48 h and evolves in three stages leading to a total resistance increase of 10%, in line with those observed for L-cysteine binding on npAu (Hengge et al^4^). The first (red) stage correlates to binding on the outer and easily accessible surface, the second stage (green) to binding in the internal structure and the third (blue) to reordering and “standing-up” of the SAM molecules accompanied by additional binding.
Subsequently, npAu/MESA sample was rinsed with water by immersion for 1 h followed by cyclic voltammetry (and concomitant resistometry) in 1M KOH (-40 to -1000 mV, 1 mVs^-1^), Figure S3 B-C. The transferred charge (in coulomb) during the reduction peak in the first cycle (red) is correlated to the simultaneous resistance decrease (∆R). From this correlation (∆R per coulomb), the initial surface coverage of MESA on npAu was determined based on the total resistance increase during absorption, following Hengge et al.^[4]^

*Preparation and purification of BM3*

All procedures and materials were those from Valikhani et al.^[5]^ Expression of P450 BM3 Z_basic2_ was done in E.coli Lemo21 cells (New England Biolabs). The preculture was grown at 37 °C and 110 rpm for 16 h in 100 mL lysogeny broth (supplemented with 0.05 mg/mL kanamycin). 2 mL of the preculture were taken to inoculate 200 mL of terrific broth media (supplemented with 0.05 mg/mL kanamycin) in 1 L flasks. To start protein expression, isoproyl β-D-1 thiogalactopyranoside (1 mM final concentration) and the heme pre-cursor 5-aminolevulenic acid (0.5 mM final concentration) was added at OD600 = 1. Incubation was done for 40 h at 18 °C (110 rpm). Afterwards, the cells were separated from the cultivation broth by centrifugation at 5000 rpm and 4 °C for 20 min, resuspended in potassium phosphate buffer and centrifuged again at 4000 rpm, at 4 °C for 15 min. After removing of the supernatant, the cell pellets were finally stored at -20 °C to enhance cell disruption.

After thawing, the cell pellet was resuspended in KPi buffer (50 mM, pH 7.5) to roughly two-fold of its original volume and disrupted by sonification (at 70% amplitude with 2 s pulse followed by 4 s pulse off, in total 5 min pulse time). The pH was adjusted to pH 7.5 using 1 M KOH. Purification was done using a pre-packed cation exchange column (HiTrap SP FF column (16 x 25 mm; 5 mL) from GE Healthcare Life Sciences). The column was equilibrated with 50mM KPi, pH 7.5, and then 5-7 mL of filtered cell-free extract was loaded onto the column. A continuous salt gradient (total volume of 75 mL, 3 mLmin^-1^ flow rate) from 0 to 100% of 50 mM KPi containing 2 M NaCl, pH 7.5, was used for protein elution. Protein eluted at 55-65%. Samples were concentrated by centrifugation in Vivaspin Turbo (Sartorius Stedim Lab Ltd), 50 kDa cut-off, to a volume of 1 mL and then resuspended with 10 mL of 50 mM KPi (pH 7.5). This step was repeated 4 times for desalting. After the final step, the samples were suspended to roughly 1 µM (130 µg/mL) and stored in aliquots of 1 mL at -20 °C.

*Quantification of enzyme concentration*

Enzyme concentration (number of active sites) was determined by CO-titration.^[6,7]^ After reducing the Fe^3+^ of the heme group to Fe^2+^ and binding of carbon monoxide (CO), the absorption peak shifts from 420 towards 450 nm. 1 mL of BM3 (in appropriate dilution in 50 mM KPi, pH 7.5) was mixed with a spatula tip of the reducing agent sodium dithionite and the reference spectrum was recorded. Immediately afterwards, the solution was gassed with CO for 30 s under the fume hood and a second absorption spectrum was recorded. From the change in peak height at 450 nm, the concentration of active BM3 was calculated with a molecular extinction coefficient of ε = 91 mM^-1^ cm^-1^.

**Supporting Figures**


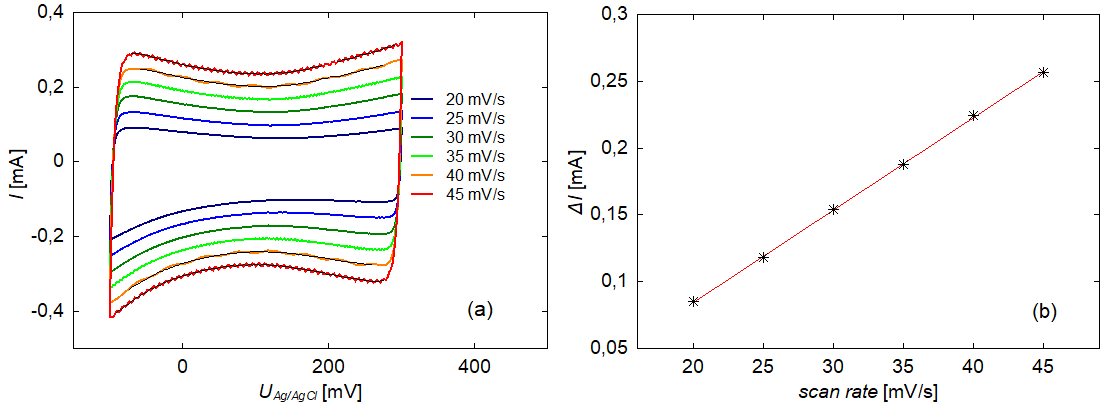


**Figure S1.** Electrochemical determination of the pore size. (a) Cyclic voltammetry in double layer regime between -100 and +300 mV at varying scan rate (20 and 45 mVs^-1^) in 0.1 M HClO_4_. (b) Background-corrected double layer current at +100 mV derived from (a) as function of scan rate. From the slope of the linear fit (shown in red) the total capacitance is determined.


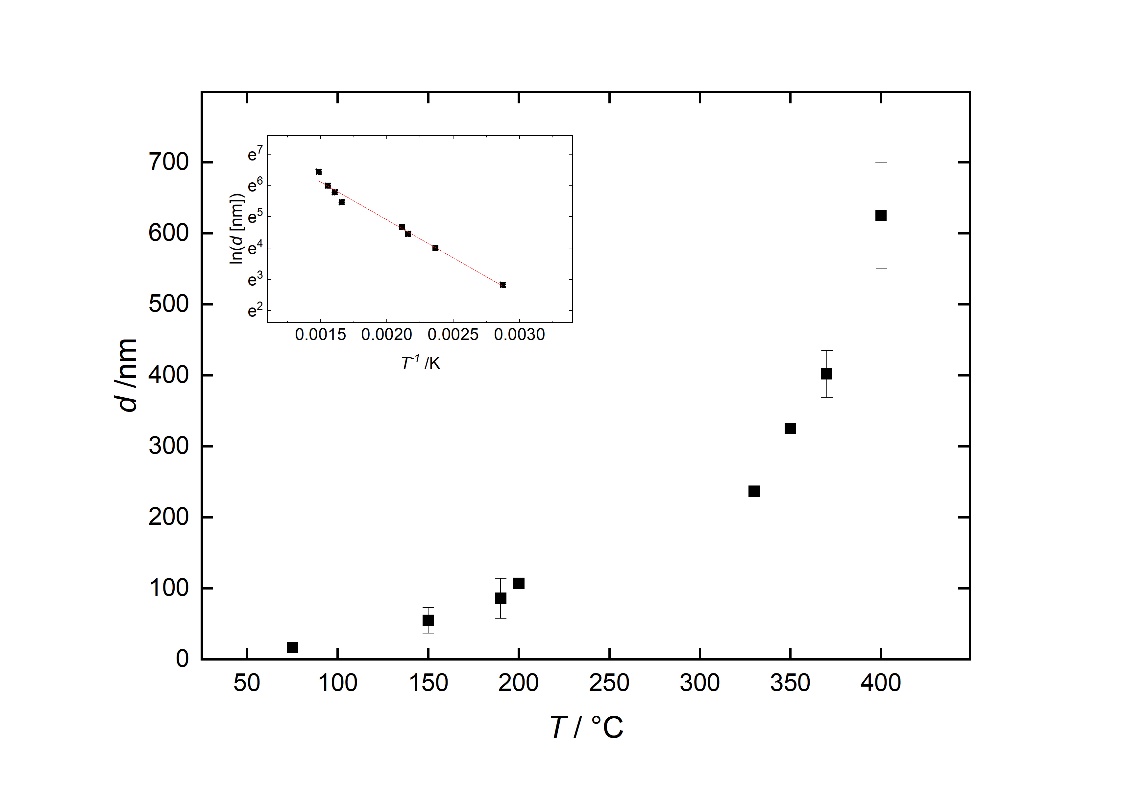


**Figure S2.** Dependence of the final pore diameter *d* of npAu on the annealing temperature *T*. One data point is the arithmetic mean of multiple samples. The error bars are calculated by the standard deviation of the resulting pore sizes. Note that a varying number of samples was available for each temperature.


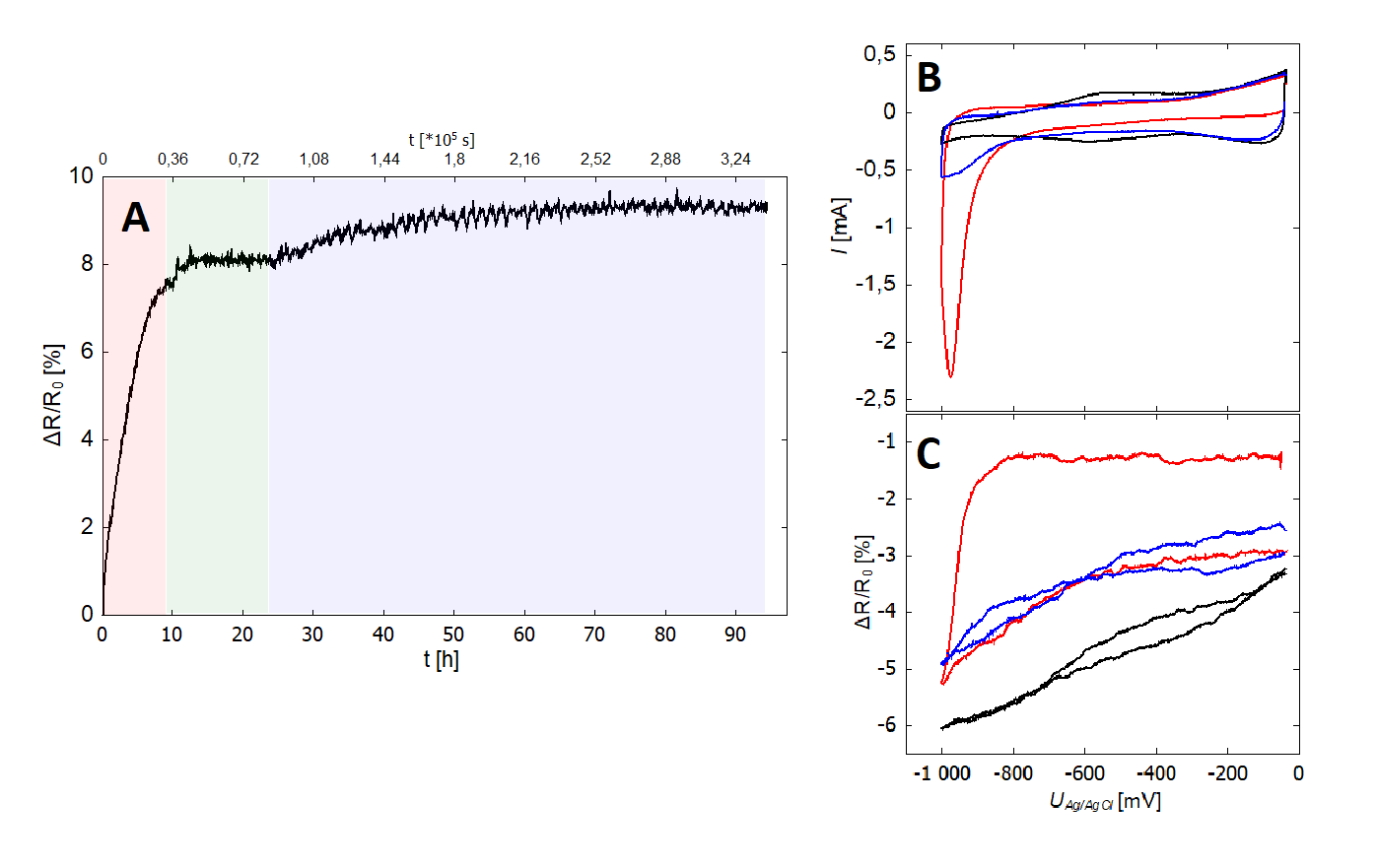


**Figure S3.** *In situ* resistometry. A) Relative change in electrical resistance during the incubation of npAu in 5 mM MESA (in distilled water). B-C) Cyclic voltammetry of npAu modified with MESA (B) and concomitant measured change in relative electrical resistance (C). The colour indicate the cycle number, red - first, blue – second, black - 10^th^ cycle.


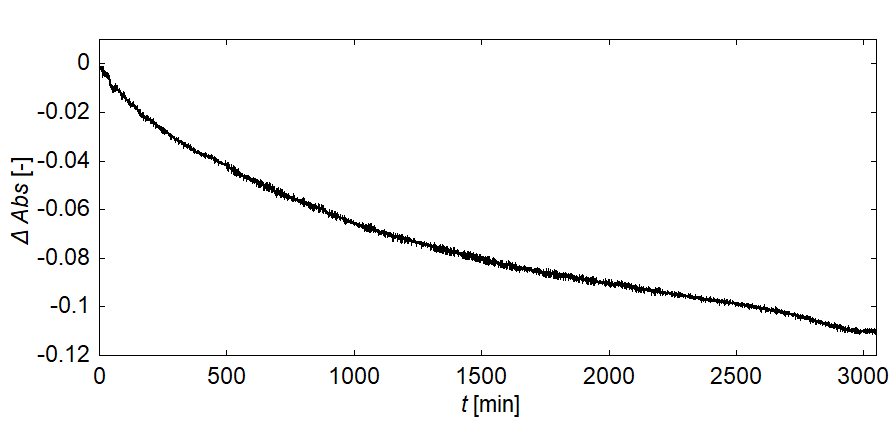


**Figure S4.** Absorbance change at 420 nm over time during the immobilization of BM3 on npAu/MESA. Absorbance is given relative to a reference solution without npAu/MESA.


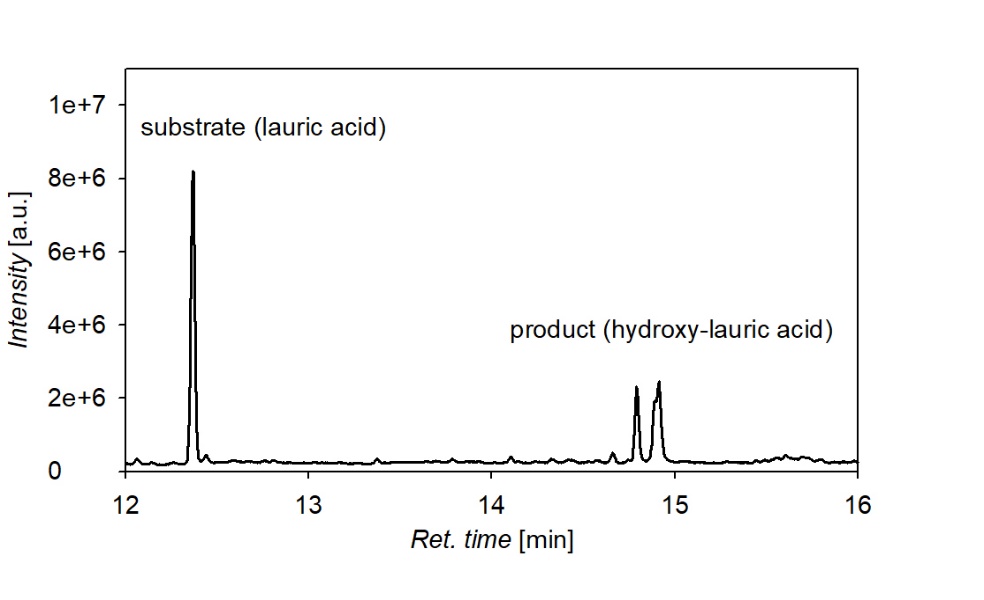


**Figure S5.** GC-FID analysis of lauric acid hydroxylation with soluble BM3. Conversion of 0.4 mM lauric acid with 0.25 µM BM3 and 0.4 mM NADPH (total volume 2 mL, 30°C).


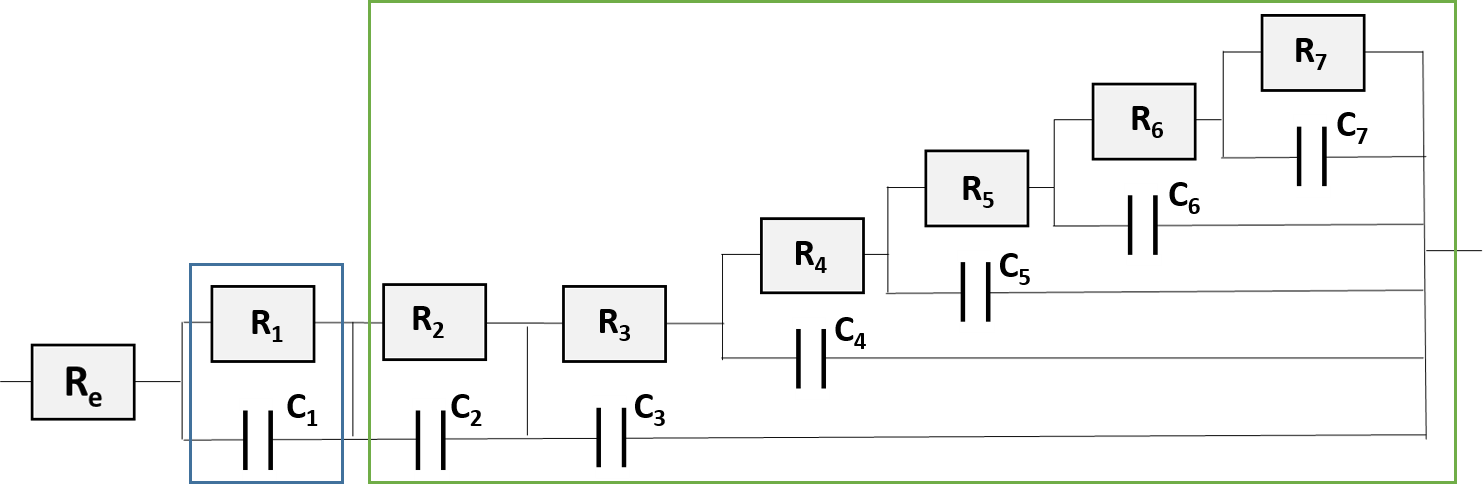


**Figure S6.** Electrical equivalent circuit used for fitting the impedance data obtained during immobilization of BM3 on npAu/MESA. R_e_ represents the electrolyte resistance, the RC element marked in blue is the impedance caused by the contact between the sample and the contacting Au-wire. The RC elements marked in green represent the porous structure as well as the surface modification and protein layer. EEC fit parameters are presented below in Table S1.


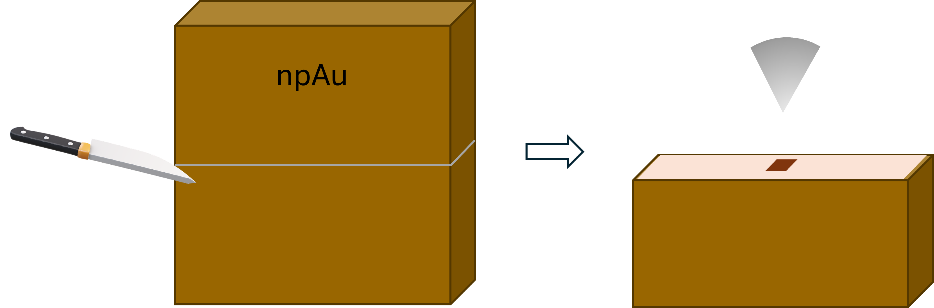


**Figure S7.** Schematic representation of the sample preparation for SEM. The npAu/MESA and npAu/MESA/BM3 sample, respectively, was cut in half with a scalpel and placed upright on a sample holder. This enabled investigation of the central area of the cross-section, depicted in dark red.


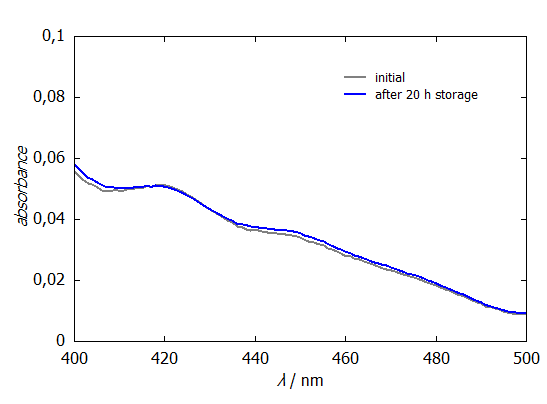


**Figure S8.** UV spectra of BM3 in solution (without npAu-electrode) during storage in 4°C. Grey: after 0 h storage, blue: after 20 h storage. 1:5 dilution in 50 mM KPi, stock solution 0.9 µM.


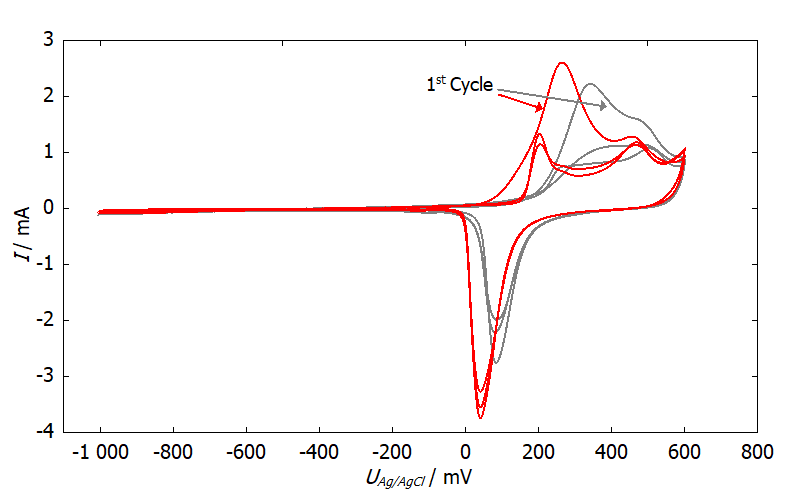


**Figure S9**. Cyclic voltammograms in 1 M KOH (5 mVs^-1^) to study the stability of the SAM after regeneration experiments of NADPH. Grey: npAu/MESA which was not used for NADPH-regeneration. Red: npAu/MESA after NADPH-regeneration.


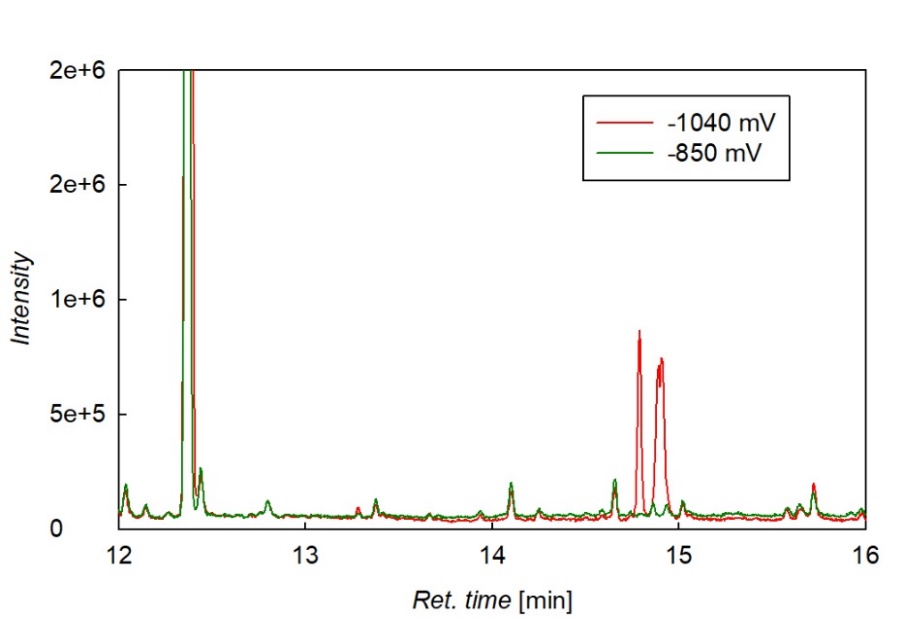


**Figure S10.** GC-FID analysis of lauric acid hydroxylation with soluble BM3. Conversion of 1.6 mM lauric acid with 0.45 µM BM3 and 1.6 mM NADP^+^ in 50 mM KPi, RT. npAu/MESA sample was used as electrode to convert NADP^+^ to NADPH, applied potentials -1040 mV (red curve) and -850 mV (green curve) for total of 3.5 h.


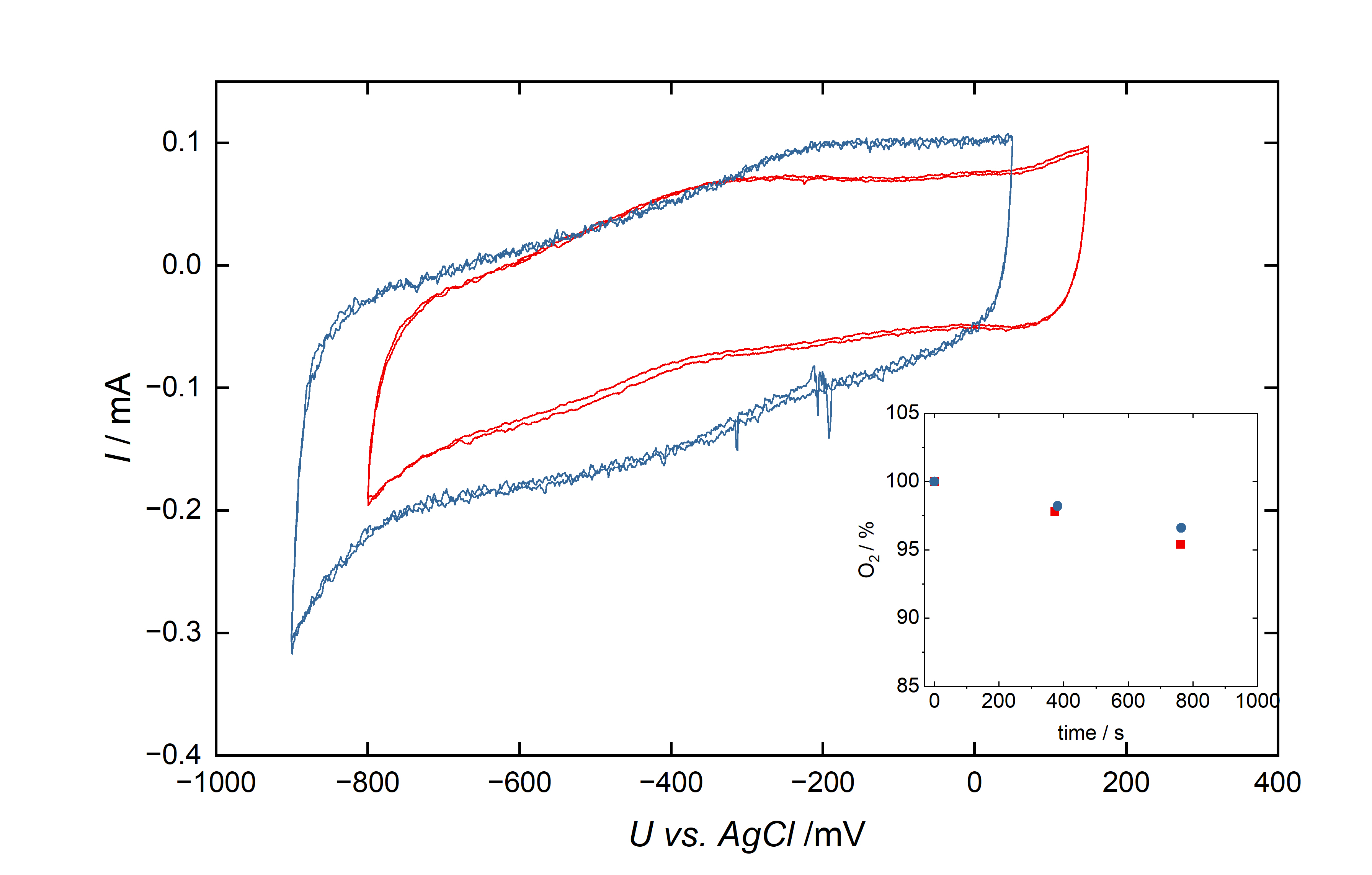


**Figure S11.** Cyclic voltammetry of npAu/MESA/BM3 to investigate DET. Blue: npAu/MESA and red: npAu/MESA/BM3 in 50mM KPi containing 80mM lauric acid (100 μM final concentration), 5 mVs^-1^, 30°C. Difference in current originates from the surface modifications and correlate to change in total capacitance. Inset: Evolution of O_2_ concentration in the reaction volume recorded at the same potential-step during 3 consecutive cycles.


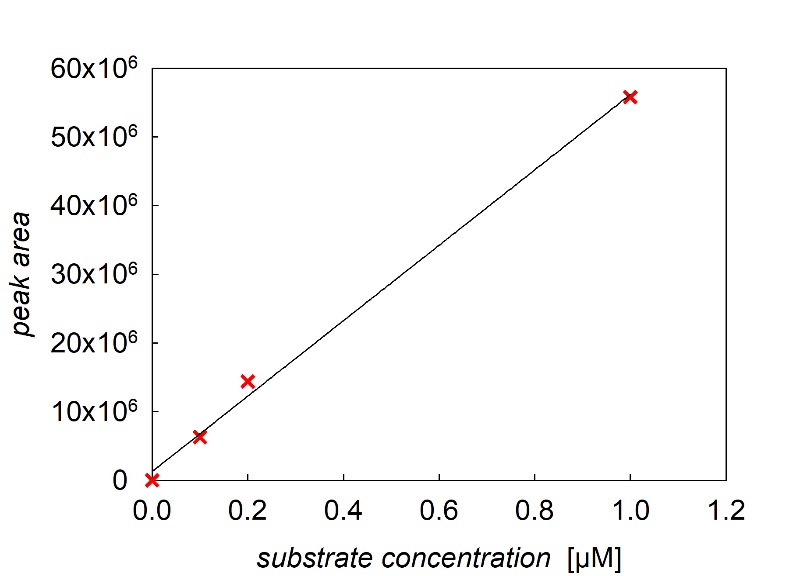


**Figure S12**. Calibration for quantitative GC analysis. The peak area was calculated from the integral of lauric acid concentration ranging from 0 to 1 µM.

**Supporting Tables**

**Table S1**. EEC fit results for the impedance spectra presented in Figure 4A and 5A. Fitting was done with NOVA software from Metrohm. Corresponding EEC is shown in Figure S6.

|  | Figure 4A | | Figure 5A | |
| --- | --- | --- | --- | --- |
|  |  | | *Nanoscale Stirring* | |
|  | First spectrum | Last spectrum | First spectrum | Last spectrum |
| R_e_ / Ω | 79.2 | 82 | 75.6 | 26.6 |
| R_1_ / Ω | 18.1 | 13.5 | 13.3 | 28.7 |
| R_2_ / Ω | 17 | 9.77 | 5.2 | 39.3 |
| R_3_ / Ω | 0.182 | 21.9 | 16.6 | 22.3 |
| R_4_ / Ω | 45.2 | 188 | 50 | 5.82 |
| R_5_ / Ω | 171 | 67200 | 1400 | 47.1 |
| R_6_ / Ω | 18.0k | 12.3M | 8520 | 12300 |
| R_7_ / Ω | 327.0k | 12.0M | 26700 | 39600 |
| C_1_ / µF | 7.66 | 4.2 | 11.7 | 15700 |
| C_2_ / µF | 61.7 | 542 | 0.066 | 0.001 |
| C_3_ / µF | 0.001 | 10.2 | 30.7 | 8.55 |
| C_4_ / µF | 276 | 855 | 1120 | 99.3 |
| C_5_ / µF | 654 | 546 | 1040 | 1100 |
| C_6_ / µF | 578 | 115 | 420 | 1.02 |
| C_7_ / µF | 80.3 | 9.53 | 864 | 714 |

**Table S2**. Fit results for peak-fitting of the Amide II peak in the GIR-IR-spectra in Figure 4D (inset). Analysis was done via OriginPro2022®.

| Model | Gaussian | | | |
| --- | --- | --- | --- | --- |
| Equation | y = y0 + A/(w*sqrt(pi/(4*ln(2)))) * exp(-4*ln(2)*(x-xc)^2/w^2) | | | |
| Assignment | α-helix | β-turns | random coils | β-sheets |
| y0 | -1.94E-5 ± 3.8E-6 | -1.93E-5 ± 3.8E-6 | -1.94E-5 ± 3.8E-6 | -1.94E-5 ± 3.8E-6 |
| xc | 1662.8 ± 0.6 | 1684.1 ± 0.8 | 1642.5 ± 0.9 | 1625.5 ± 4.0 |
| A | 0.17 ± 0.03 | 0.13 ± 0.01 | 0.06 ± 0.03 | 0.02 ± 0.01 |
| w | 28± 2 | 30 ± 1 | 24 ± 3 | 24± 2 |
| Χ^2^ (reduced) | 1.16E-10 | | | |
| R^2^ | 0.99999 | | | |
| R^2^ (corr.) | 0.99998 | | | |

**References**

[1] E. Detsi, E. Jong, A. Zinchenko, Z. Vuković, I. Vuković, S. Punzhin, K. Loos, G. Brinke, H. A. Raedt, P. R. Onck, J. T. M. Hosson, *Acta Mater.* **2011**, *59*, 7488

[2] C. Lakshmanan, R. Viswanath, S. Polaki, R. Rajaraman, *AIP Conf. Proc.* **2015**, *1665*,
 140033.

[3] F. Habashi, *Encyclopedia of Metalloproteins*, Springer New York, New York, NY, USA
 **2013**.

[4] E. Hengge, E.-M. Steyskal, R. Bachler, A. Dennig, B. Nidetzky, R. Würschum, *Beilstein J. Nanotechnol.* **2019**, *10*, 22752279.

[5] D. Valikhani, J. M. Bolivar, A. Denni, B. Nidetzky, *Biotechnol. Bioeng.* **2018**, *115*, 2416.

[6] M. B. Buergler, A. Dennig, B. Nidetzky, *Biotechnol. Bioeng.* **2020**, *117*, 2377.

[7] A. Dennig, N. Lülsdorf, H. Liu, U. Schwaneberg, *Angew. Chem., Int. Ed.* **2013**, *52*, 8459.
